# Gene expression dynamics in fibroblasts during early-stage murine pancreatic carcinogenesis

**DOI:** 10.1016/j.isci.2024.111572

**Published:** 2024-12-10

**Authors:** Nupur Ohri, Johanna Häußler, Nino Javakhishvili, David Vieweg, Anais Zourelidis, Bogusz Trojanowicz, Monika Haemmerle, Irene Esposito, Markus Glaß, Yoshiaki Sunami, Jörg Kleeff

**Affiliations:** 1 Department of Visceral, Vascular and Endocrine Surgery, Martin-Luther-University Halle-Wittenberg, University Medical Center Halle, 06120 Halle (Saale), Germany; 2 Institute of Medical and Public Health Research, Ilia State University, Tbilisi 0162, Georgia; 3 Institute of Pathology, Martin-Luther-University Halle-Wittenberg, University Medical Center Halle, 06112 Halle (Saale), Germany; 4 Institute of Pathology, Medical Faculty and University Hospital Düsseldorf, Heinrich-Heine-University, 40225 Düsseldorf, Germany; 5 Institute of Molecular Medicine, Martin-Luther-University Halle-Wittenberg, 06120 Halle (Saale), Germany

**Keywords:** Biological sciences, Genetics, Cancer

## Abstract

Pancreatic ductal adenocarcinoma (PDAC) is characterized by aggressive growth and metastasis, partly driven by fibroblast-mediated stromal interactions. Using RNA sequencing of fibroblasts from early-stage KPC mouse models, we identified significant upregulation of genes involved in adipogenesis, fatty acid metabolism, and the ROS pathway. ANGPTL4, a key adipogenesis regulator, was highly expressed in fibroblasts and promoted pancreatic cancer cell proliferation and migration through paracrine signaling. Notably, cancer cell-driven paracrine signals appear to regulate ANGPTL4 expression in fibroblasts, suggesting that ANGPTL4 may act as a reciprocal factor in a feedback loop that enhances tumor progression. LAMA2, an extracellular matrix gene with reduced expression, suppressed pancreatic cancer cell migration, proliferation, and invasion. This study provides the temporal transcriptional analysis of fibroblast subtypes during early PDAC, highlighting the roles of metabolic reprogramming and ECM remodeling in shaping the tumor microenvironment and identifying potential therapeutic targets.

## Highlights

• ANGPTL4 drives tumor progression via a paracrine feedback loop in PDAC fibroblasts
• LAMA2 suppresses cancer cell migration, proliferation, and invasion in PDAC
• Metabolic reprogramming in fibroblasts shapes the early PDAC tumor microenvironment
• Temporal transcriptomic analysis reveals fibroblast roles in early PDAC progression

## Graphical abstract

**Figure:**
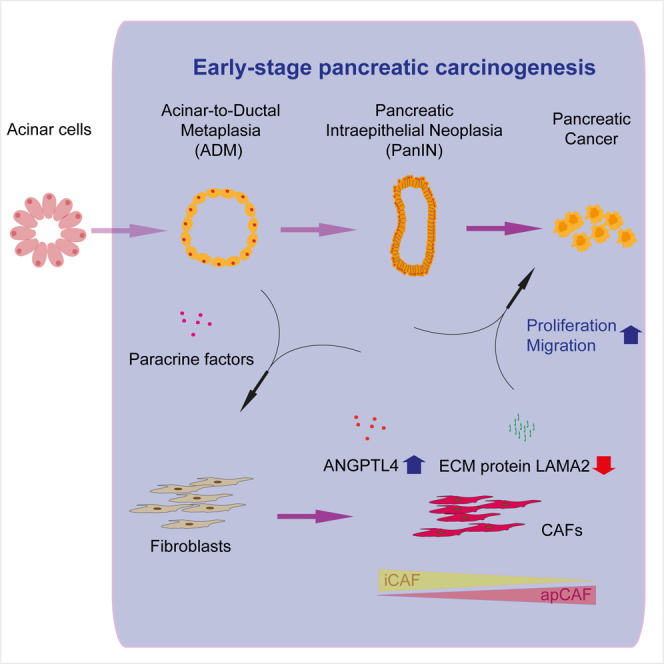

## Introduction

Pancreatic ductal adenocarcinoma (PDAC) represents an exceptionally lethal malignancy, hallmarked by its propensity for aggressive local tumor growth and metastatic dissemination. Despite the substantial strides made in cancer research and management, the current overall relative 5-year survival rate for PDAC is dismal, hovering around 12%, placing it among the most fatal cancers known to date.^1^ Moreover, PDAC has been predicted to emerge as the second most prevalent cause of cancer-related deaths by 2030.^2^ A significant number of PDAC cases are diagnosed at locally advanced stages and/or with distant metastatic dissemination at the time of diagnosis. Consequently, potentially curative surgical interventions are not achievable for the majority of patients, highlighting the pressing need for innovative diagnostic and therapeutic strategies that can address the aggressive nature of this disease and improve the survival of patients with PDAC.^3^

The local tumor microenvironment of PDAC has garnered significant attention in recent years. Studies have revealed that the abnormal dynamics of the extracellular matrix (ECM) within the tumor microenvironment play a crucial role in promoting early metastasis and drug resistance of PDAC cells. Thus, the ECM influences the signaling pathways that regulate cell survival, proliferation, and drug resistance, further contributing to the aggressive and treatment-resistant phenotype of PDAC.^4^ It is becoming increasingly evident that fibroblasts are the predominant cells present in the stroma of the PDAC tumor microenvironment and are responsible for the elaboration of various components of the extracellular matrix, including collagens, proteolytic enzymes, proteolytic inhibitors, growth factors and structural proteoglycans, thereby shaping the tumor microenvironment and influencing tumor progression.^5^

The fibroblasts within the tumor stroma acquire a modified phenotype, similar to fibroblasts associated with wound healing, and termed as CAFs (cancer-associated fibroblasts).^6^ Under normal circumstances, pancreatic stellate cells (PSCs) which are the resident fibroblastic cells of the pancreas exist as quiescent cells characterized by the persistence of vitamin A-rich lipid droplets. Under stimuli generated by tissue injury and stress, as for example in chronic pancreatitis, and PDAC, PSCs activate into a myofibroblast-like phenotype^7^ and these lipid droplets disappear from the cytosol. CAFs not only arise from activated PSCs but also from other fibroblasts of the pancreas or being recruited from the bone marrow. CAFs can also arise from various other cell types, such as mesenchymal stem cells,^8^ endothelial cells,^9^ adipocytes,^10^ epithelial cells,^11^ due to cues from pre-neoplastic cells.^12^^,^^13^

In the progression of PDAC, activated PSCs/CAFs are thought to develop an "unholy alliance”^14^ with cancer cells and acquire tumor-permissive properties, such as facilitating metastasis, immune evasion, extracellular matrix remodeling, metabolic support, and resistance to therapy.^15^ It has become clear, however, that CAFs are not only tumor promoting but also tumor restraining depending on the spatial and temporal tumor development as well as on the specific CAF subtype.^16^^,^^17^^,^^18^^,^^19^ Thus, recent studies utilizing genetically engineered mouse models of PDAC and single-cell RNA sequencing (scRNA-seq) identified various subtypes of cancer-associated fibroblasts (CAFs), including myofibroblastic CAFs (myCAFs), antigen-presenting CAFs (apCAFs), which exhibited immunosuppressive properties, and inflammatory CAFs (iCAFs).^20^^,^^21^ The identification of distinct CAF subtypes with opposing effects on tumor growth highlights the heterogeneity of CAFs in PDAC. This heterogeneity presents a promising avenue for the development of targeted therapies that could effectively treat PDAC. The potential to selectively target CAF offers a unique opportunity to improve current treatment options for PDAC and improve patient outcomes.^22^

While the infiltration and activation of PSCs in proximity to early precursor lesions is a well-known phenomenon,^23^ the molecular mechanisms underlying fibroblast activation and their precise role in mediating communication with cancer cells during PDAC initiation and progression remain unknown. These observations underscore the importance of further investigation into the mechanisms driving PSC activation and their interplay with cancer cells, which may lead to the development of new therapeutic strategies for PDAC.

To enhance our comprehension of the molecular behavior of fibroblasts in PDAC and assess the crucial signaling pathways involved in the interaction of pre-neoplastic cells and PSCs within the complex stromal environment, we aim to investigate the alterations in the transcriptional profile of these fibroblasts during the initial phase of carcinogenesis. The objective is to uncover the molecular signatures that may aid in the early diagnostics of PDAC using RNA sequencing of fibroblasts obtained from a well-established genetically engineered mouse model of PDAC (KPC: *Ptf1a/p48-Cre; lox-stop-lox-Kras*^*G12D/+*^*; lox-stop-lox-Trp53*^*R172H/+*^) at distinct time points that correspond to precursor and the early, pre-invasive stage of PDAC. The KPC model exhibits mutation in oncogenic *KRAS* and *Trp53* under the *Ptf1a* promotor^24^ and is characterized by the development of precursor lesions at around 8–10 weeks of age, followed by a tendency to develop invasive pancreatic adenocarcinoma mirroring the clinical features observed in humans within 14–16 weeks.^25^

## Results

### The transcriptional profile of murine fibroblasts in early pancreatic carcinogenesis

**Figure 1:**
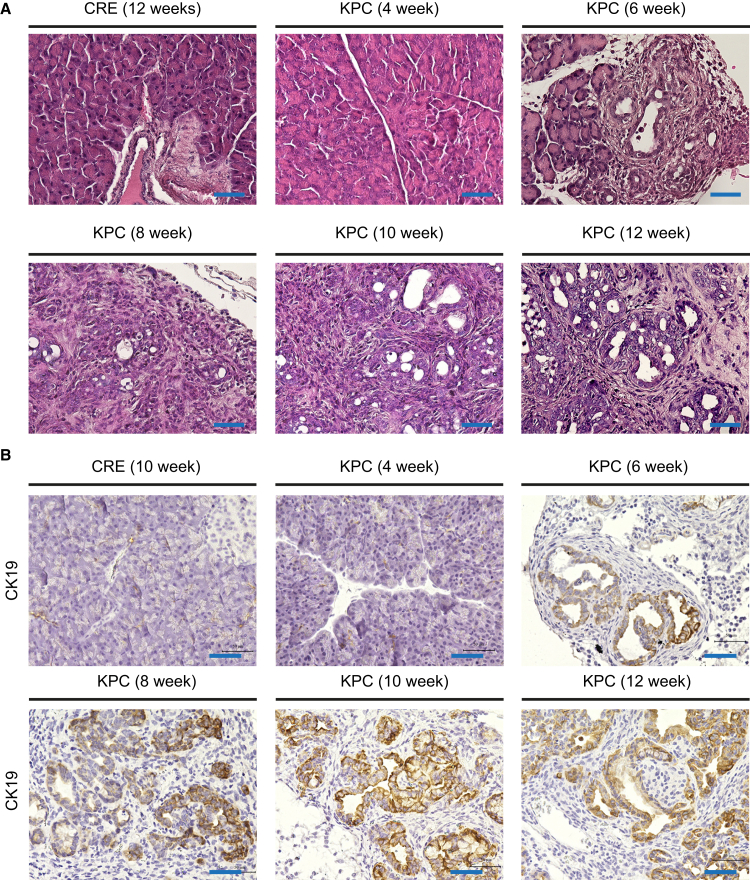
Aciar-to-ductal metaplasia (ADM) and pancreatic intraepithelial neoplasia (PanIN) formation in KPC (*Ptf1a/p48-Cre; lox-stop-lox-Kras*^*G12D/+*^*; lox-stop-lox-Trp53*^*R172H/+*^) mice (A) H&E staining reveals the pathological conditions of CRE aged 12 weeks and KPC pancreatic tissue of mice aged 4, 6, 8, 10, and 12 weeks. Preneoplastic lesions were observed starting at 6 weeks and increased with age (Bar: 50 μm). (B) Immunohistochemistry analyses for a ductal marker CK19 (Bar: 50 μm).

**Figure 2:**
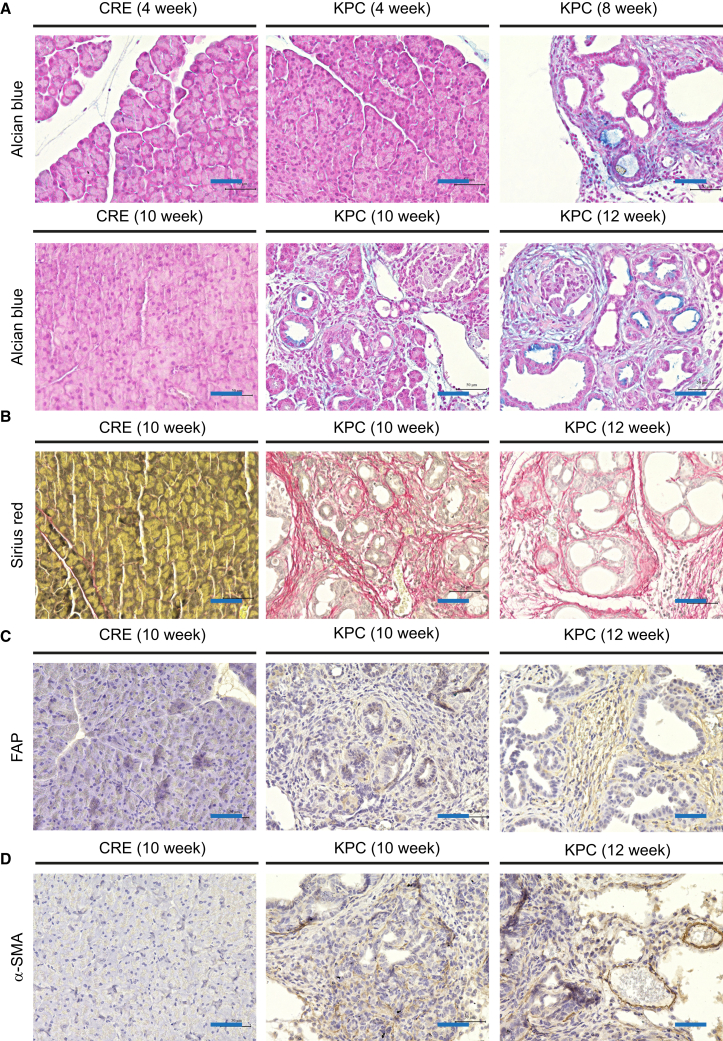
Elevated mucin secretion and fibrotic reaction in KPC mice (A) Alcian blue staining was performed in pancreatic tissue from CRE mice at ages 4 and 10 weeks, and KPC mice at ages 4, 6, 10, and 12 weeks. Mucin positivity was observed at 6 weeks (Bar: 50 μm). (B) Sirius red staining for evaluating collagen accumulation in the pancreas of CRE mice at 10 weeks and KPC mice at 10 and 12 weeks. (C) Fibroblast activation protein (FAP) staining, a marker for fibroblasts, was conducted on pancreatic tissue from CRE mice at 10 weeks and KPC mice at 10 and 12 weeks. (D) α smooth muscle actin (α-SMA) staining was performed as an additional fibroblast marker for pancreatic tissue from CRE mice at 10 weeks and KPC mice at 10 and 12 weeks.

To investigate the role of fibroblasts in the early-stage of pancreatic carcinogenesis, specifically to analyze transcriptomic dynamics, RNA sequencing was conducted on fibroblasts isolated from KPC (*Ptf1a/p48-CRE; lox-stop-lox-Kras*^*G12D/+*^*; lox-stop-lox-Trp53*^*R172H/+*^) and CRE (*Ptf1a/p48-CRE*) mice at age 4, 6, 8, 10, and 12 weeks (at passage 4). Hematoxylin and eosin staining confirmed the presence of Acinar-to-Ductal Metaplasia (ADM) (starting at 6 weeks) and PanIN lesions (starting at week 10 weeks) (Figure 1A). Additionally, we observed robust CK19 expression, a ductal marker, in the KPC pancreata (Figure 1B). Histological analyses using Alcian blue and Sirius red staining revealed a fibrotic reaction in KPC mice (Figures 2A and 2B). Furthermore, we detected fibroblast marker proteins, fibroblast activation protein (FAP) and α-smooth muscle actin (α-SMA) (Figures 2C and 2D). Therefore, this time frame (4–12 weeks) was considered highly relevant to analyze ADM/PanIN-associated fibroblasts during early pancreatic carcinogenesis.

**Figure 3:**
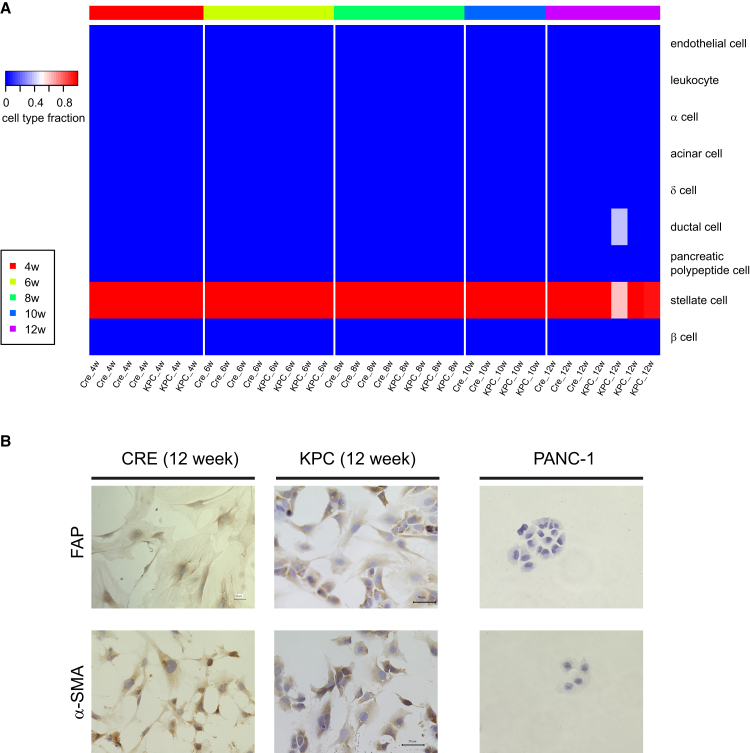
Characterization of primary isolated fibroblasts from CRE and KPC mice (A) CIBERSORTx-based cell type deconvolution demonstrates the purity of pancreatic fibroblasts in CRE and KPC mice across the 4–12-week time points. (B) Immunocytochemistry of FAP and α-SMA in fibroblasts from 12-week-old CRE or KPC mice. PANC-1 cells served as a negative control.

Cell type deconvolution of RNA-seq data generated from primary isolated fibroblasts revealed RNA expression profiles reminiscent of pancreatic stellate cells in all but one sample. However, in fibroblasts from one 12-week-old KPC mouse, we observed a ductal cell-weighted profile (Figure 3A). To avoid potential contamination and misinterpretation of data, we omitted this sample from further analyses. Immunocytochemistry for FAP and α-SMA on primary isolated cells confirmed positive staining in fibroblasts from both CRE and KPC mice, whereas PANC-1 pancreatic cancer cells showed no staining (Figure 3B).

### Temporal changes of transcriptional regulation in fibroblasts from CRE versus KPC mice

**Figure 4:**
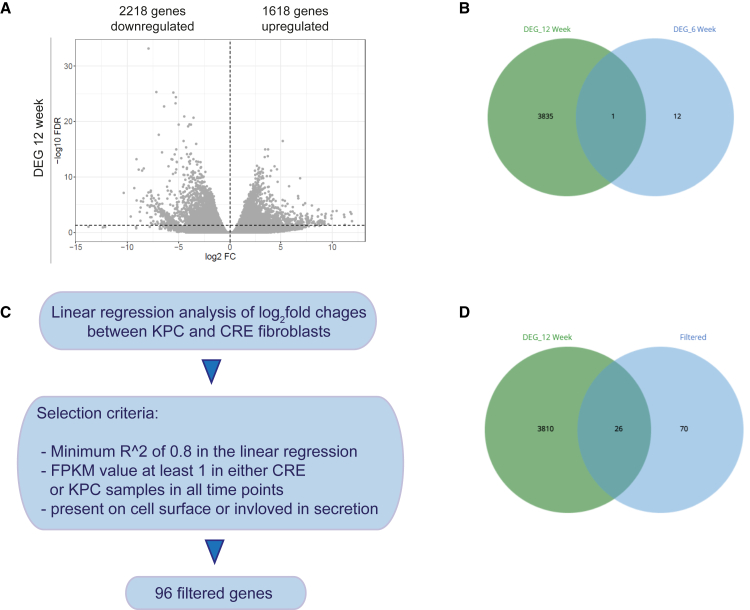
Differentially expressed genes (DEGs) in KPC fibroblasts (A) DEG in KPC fibroblasts compared to CRE fibroblasts at the 12-week time point. (B) Venn diagram showing the presence of one overlapping protein coding gene between time points 6 and 12 weeks. (C) Schematic representation for the selection of 96 genes. (D) The Venn diagram illustrates the presence of 26 target genes meeting the specified selection criteria (section-of results) and identified as differentially expressed at the 12-week time point. These targets may potentially play a role in paracrine communication between fibroblast and preneoplastic cells in PDAC.

A differential gene expression analysis was conducted in fibroblasts to identify and analyze the dynamics in transcriptional profiling between CRE and KPC mice. In adhering to FDR <0.05 and a log_2_ fold change cut-off of |1|, no significantly differentially expressed genes (DEGs) were found at 4, 8, and 10 weeks. At 6 weeks, 13 DEGs (8 upregulated and 5 downregulated) were observed. For the 12-week time point, a total of 1618 genes were found to be upregulated, while 2218 genes were identified as downregulated (in total 3836 genes). This suggests that differences in gene expression between CRE and KPC fibroblasts are more pronounced at the 12-week time point (Figures 4A and 4B).

We observed only a few DEGs between the 4 and 10-week time points. However, we hypothesized that there might be genes that were not identified as significant DEGs at the early time points but showed a continuous increase or decrease in expression throughout the entire time course. To identify such genes, we performed a linear regression analysis of log2 fold changes between KPC and CRE samples. For further investigation, we selected protein-coding genes based on the following criteria: (a) a minimum coefficient of determination (Rˆ2) of 0.8 in the linear regression, and (b) sufficient expression, with an average Fragments Per Kilobase of exon per Million reads mapped (FPKM) value of at least 1 in either CRE or KPC samples in all time points (Figure 4C). As fibroblasts secrete factors or interact directly with other cell types, we utilized Gene Ontology annotation^26^ to select factors known to be present on the cell surface or involved in secretion. We identified 96 factors among which 26 genes were also identified as relevant DEGs at the 12-week time point (Figure 4D and Tables S1–S5).

### Significant gene expression alteration for cell cycle pathway, Myc targets, and ECM-associated genes in KPC fibroblasts

**Figure 5:**
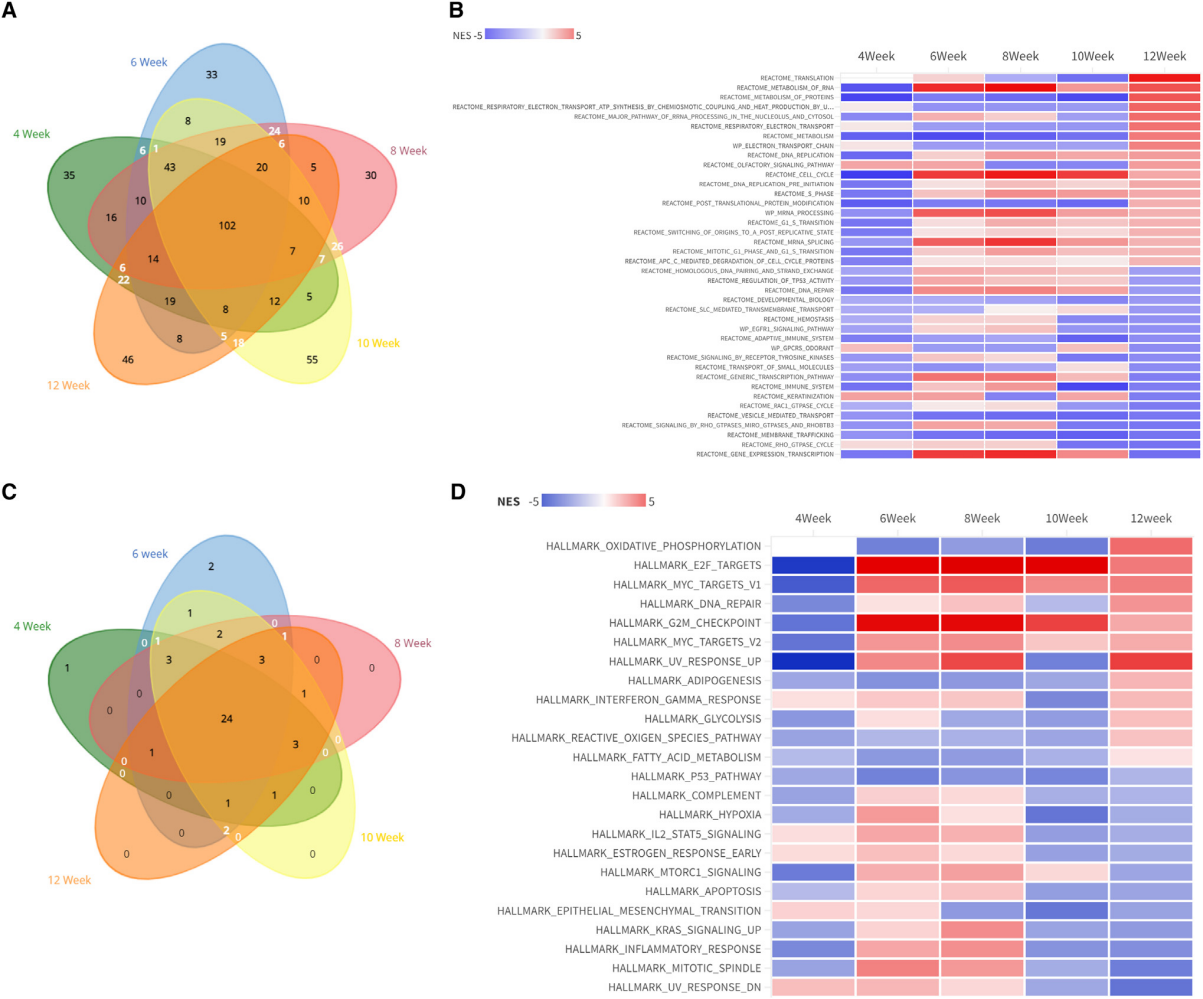
Gene expression alteration in biological pathways in KPC fibroblasts (A) The Venn diagram shows 102 canonical pathways exhibited significant enrichment in KPC fibroblast as compared to CRE in all time points (4–12 weeks). (B) Heatmap shows the top 20 positively and negatively enriched canonical pathways based on decreasing order of normalized enrichment scores (NES) of 12 weeks. (C) The Venn diagram shows 24 hallmark gene sets exhibited significant enrichment in KPC fibroblast as compared to CRE in all time points (4–12 weeks). (D) The heatmap presents the normalized enrichment scores (NES) of 24 hallmark gene sets that exhibited consistency across all examined time points.

To better understand alterations in biological pathways and key biological processes in KPC fibroblasts, we performed gene set enrichment analysis using 1691 canonical pathways and 50 hallmark gene sets from MSigDB.^27^ We observed significant enrichment (padj <0.05) of 397, 394, 429, 429, and 395 pathways in KPC-fibroblasts at time points 4, 6, 8, 10, 12 weeks respectively, as compared to CRE control (Figure 5A). Notably, a subset of 102 pathways exhibited enrichment in KPC-fibroblasts across all time points (Figure 5A). To visualize the enrichment patterns, heatmaps with the top 20 positively and negatively enriched pathways were generated based on the decreasing order of NES (Normalized Enrichment Score) values over 12 weeks (Figure 5B). We found that 24 out of 50 important gene sets remained consistent across the entire duration (4–12 weeks) (Figure 5C). Moreover, gene sets associated with the cell cycle, such as E2F targets, G2M checkpoints, and Myc targets, exhibited a continuous upward trend across all time points and an upward trend in gene sets related to adipogenesis, reactive oxygen species, and fatty acid metabolism were exclusively observed at the 12-week mark (Figure 5D).

### Continuous up-regulation of angiopoietin-like factor 4 in KPC fibroblasts during early-stage pancreatic carcinogenesis

**Figure 6:**
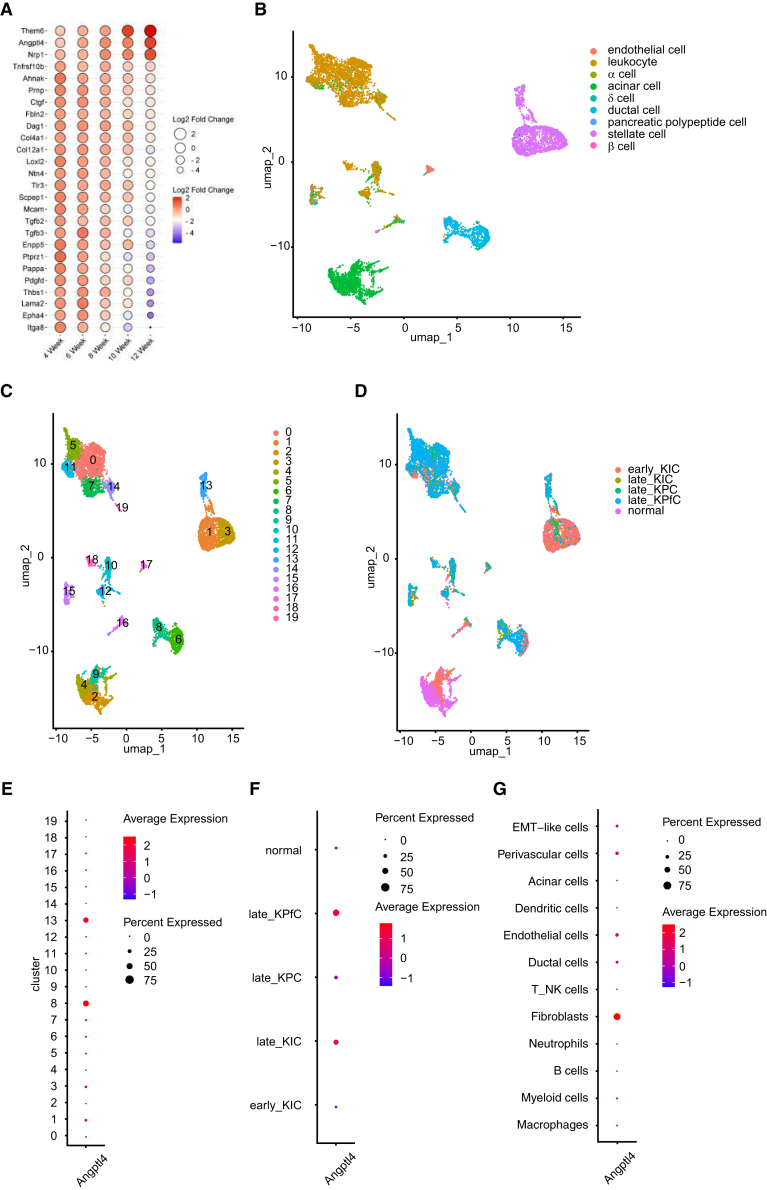
Identification of 26 continuously increased or decreased genes throughout the entire time course - especially the *Atgptl4* gene (A) Bubble plot reveals expression profile of selected 26 target-genes against log2Fold changes across all time points, 4–12 weeks. (B) UMAP plot based on scRNA-seq data from Hosein et al., showing distinct cell populations. (C) Visualization of 19 clusters identified in the UMAP analysis. (D) Gene expression status across different stages and pancreatic cancer mouse models, highlighting variations in distinct tissue compartments. (E) Expression of *Angptl4* across the 19 different clusters. (F) *Angptl4* expression within fibroblast clusters (clusters 1, 3, and 13) across different pancreatic cancer mouse models at distinct time points (early: 40 days, late 60 days). (G) *Angptl4* expression in various cell types within KPC mice based on scRNA-seq data from Elyada et al.

Among the 26 target genes selected based on the aforementioned criteria (Figure 4D and Tables S1–S5), 3 of these 26 genes - Angiopoietin-like factor 4 (*Angptl4*), Neuropilin 1 (*Nrp1*), and Thioesterase 6 (*Them6*) showed continuous up-regulation during the time course, while 23 of the 26 genes exhibited continuous down-regulation (Figure 6A). Notably, one of these genes, *Angptl4*, was found to be associated with adipogenesis. Previous studies have shown ANGPTL4, through its regulation by short chain fatty acid and peroxisome proliferator-activated receptor γ (PPARγ), may play a role in the progression of colon adenocarcinoma.^28^ Among the 26 narrowed-down targets in our dataset, several genes associated with the ECM, according to Gene Ontology, were downregulated. These genes include laminin, alpha 2 (*Lama2*), thrombospondin-1 (*Thbs1*), protein tyrosine phosphatase receptor type Z, polypeptide 1 (*Ptprz1*), transforming growth factor, beta-2, and -3 (*Tgfb*2 and *Tgfb3*), collagen 12a1 (*Col12a1*), netrin 4 (*Ntn4*), *Loxl2* (lysine oxidase-like 2), dystroglycan 1 (*Dag1*), and fibulin 2 (*Fbln2*) (Figure 6A).

A previous single-cell RNA-sequencing (scRNA-seq) study profiled cell heterogeneity during different stages of pancreatic cancer progression across various genetically engineered mouse models, namely KIC (*Ptf1a/p48-Cre; lox-stop-lox-Kras*^*G12D/+*^*; Ink4a*^*lox/lox*^), KPC, and KPfC (*Pdx1-Cre; lox-stop-lox-Kras*^*G12D/+*^*; Trp53*^*lox/lox*^).^29^ To extend these findings, we processed scRNA-seq data from those models and identified 19 distinct cell clusters, including 3 fibroblast clusters (clusters 1, 3, and 13) (Figures 6B and 6C). Fibroblasts from early-stage (40-day-old) KIC mice predominantly occupied clusters 1 and 3, whereas fibroblasts from late-stage (60-day-old) KIC, KPC, and KPfC models formed cluster 13 (Figure 6D). *Angptl4* expression was prominent in ductal cells (cluster 8) and fibroblasts (cluster 13) (Figures 6E and S1). Notably, fibroblasts from late-stage KPfC and KIC models exhibited higher *Angptl4* expression than early-stage KIC or control models (Figures 6F and S2), while late-stage KPC fibroblasts did not display elevated *Angptl4* expression (Figure 6F). However, analysis of scRNA-seq data from Elyada et al.^20^ confirmed fibroblast-enriched expression of *Agnptl4* in KPC model (Figures 6G and S3).

**Figure 7:**
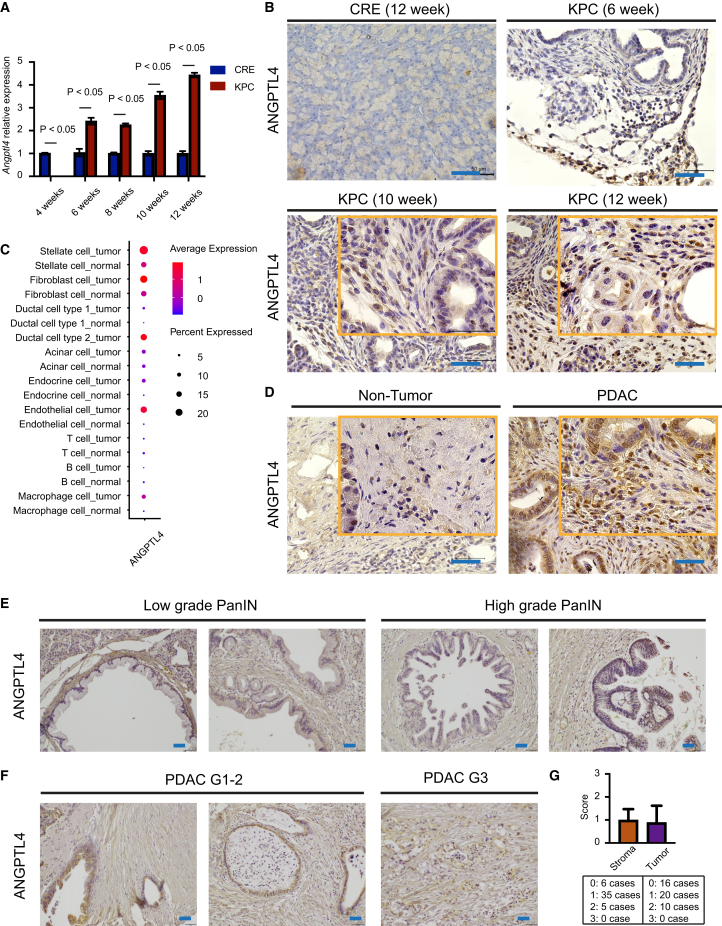
ANGPTL4 expression is elevated and more abundant in pancreatic tumors (A) RT-qPCR experiments to validate the upward expression of *Angptl4* in KPC-fibroblast as compared to CRE-fibroblast across time points. Data are represented as mean ± SD. (B) Immunohistochemistry conducted on CRE and KPC murine pancreatic tissues to show higher expression of ANGPTL4 in 10 and 12 weeks (Bar: 50 μm). (C) Analysis with human scRNA-seq data from Peng et al. revealing elevated *ANGPTL4* expression in tumor tissues. (D) Immunohistochemistry on representative human pancreatic tissues (non-tumor and PDAC) indicating higher ANGPTL4 expression in PDAC (Bar: 50 μm). (E) Immunohistochemistry on a pancreatic tissue microarray on low-grade and high-grade PanIN samples (Bar: 50 μm). (F) Immunohistochemistry conducted on a PDAC tissue microarray (G1-2, and G3) (Bar: 50 μm). (G) Staining intensity of ANGPTL4 in the stromal region and tumor cells on PDAC tissue microarray (total: *n* = 46) with scoring as follows: 0 = no staining, 1 = weak, 2 = moderate, 3 = strong. Data are represented as mean ± SD.

We observed in our mouse model that *Angptl4* expression in KPC fibroblasts was higher than in CRE control fibroblasts (*p < 0.05*) (Figure 7A). Furthermore, ANGPTL4 staining was observed in the pancreas of 10- or 12-week-old KPC mice, but not in the pancreas of 6-week-old KPC mice or in age-matched CRE control (Figure 7B). Further analysis of scRNA-seq data of human PDAC and healthy control samples (described in Peng et al.^30^) revealed prominent *ANGPTL4* expression in stellate cells and fibroblasts from healthy control samples. In human, PDAC, *ANGPTL4* expression was notably increased in stellate cells, fibroblasts, and ductal and endothelial cells (Figures 7C and S4). Immunohistochemical analysis of ANGPTL4 in human pancreatic cancer samples showed broad positivity (Figure 7D). In tissue microarray analysis, ANGPTL4 staining in PanIN tissue displayed weak stromal expression without significant differences between high- and low-grade PanIN lesions (Figure 7E). However, ANGPTL4 remained positive in PDAC stromal lesions, with additional positivity observed in endothelial cells (Figures 7F and 7G).

In conclusion, our findings demonstrate a consistent up-regulation of *Angptl4* in stromal fibroblasts during early-stage pancreatic carcinogenesis in KPC mice and human pancreatic cancer. The tissue microarray analysis confirmed stromal ANGPTL4 expression independent of PanIN stage.

### Expression of *Angptl4* in fibroblasts tends to be associated with expression of antigen-presenting cancer-associated fibroblast markers

**Figure 8:**
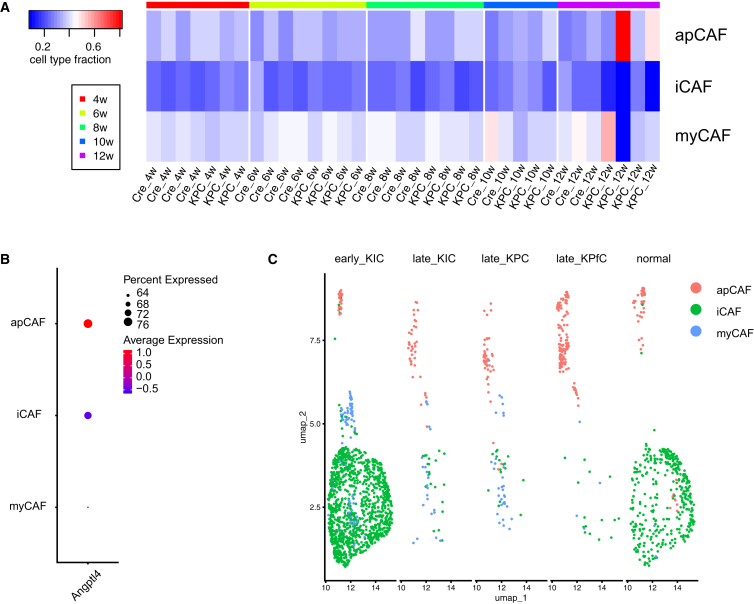
Expression of *Angptl4* in KPC fibroblasts correlates with apCAF marker expression (A) Cell type deconvolution using CIBERSORTx to reveal subtypes of CAF (myCAFs, apCAF, iCAFs) in fibroblast from CRE and KPC mice pancreas spanning the 4–12-week time period. (B) Analysis of *Angptl4* within CAF subtypes based on fibroblast scRNA-seq data from Elyada et al. (C) Distribution of CAF subtypes in various pancreatic cancer mouse models at different time points (early: 40 days, late 60 days) based on scRNA-seq data from Hosein et al.

To investigate whether there are any significant similarities between the KPC fibroblasts and the previously described CAF subtypes, we employed cell type deconvolution to assess CAF subtype proportions (apCAF, myCAF, iCAF) in CRE and KPC fibroblast samples over 4–12 weeks. While no significant differences were observed between the two groups, i.e., CRE and KPC-fibroblasts in terms of CAF subtype enrichment, there was a tendency toward apCAF-weighted enrichment in KPC fibroblasts at the 12-week time point (Figure 8A).

We further examined scRNA-seq data from Elyada et al.^20^ *Angptl4* expression was predominantly associated with the apCAF subtype (Figures 8B and S5). Additionally, analysis of fibroblasts scRNA-seq data from Hosein et al.^29^ of early and late KIC models revealed that iCAF subtypes were dominant at early-stages, but their proportions markedly decreased over time. In contrast, apCAF marker expression became more prominent in late KPfC fibroblasts (Figure 8C).

Taken together, our findings suggest that the proportions of pancreatic CAF subtypes exhibit dynamic changes over time in different mouse models, and *Angptl4* expression in fibroblasts is preferentially associated with apCAF markers.

### Over-expression of ANGPTL4 in fibroblasts induces pancreatic cancer cell proliferation and migration in paracrine manner, but not in an autocrine manner

**Figure 9:**
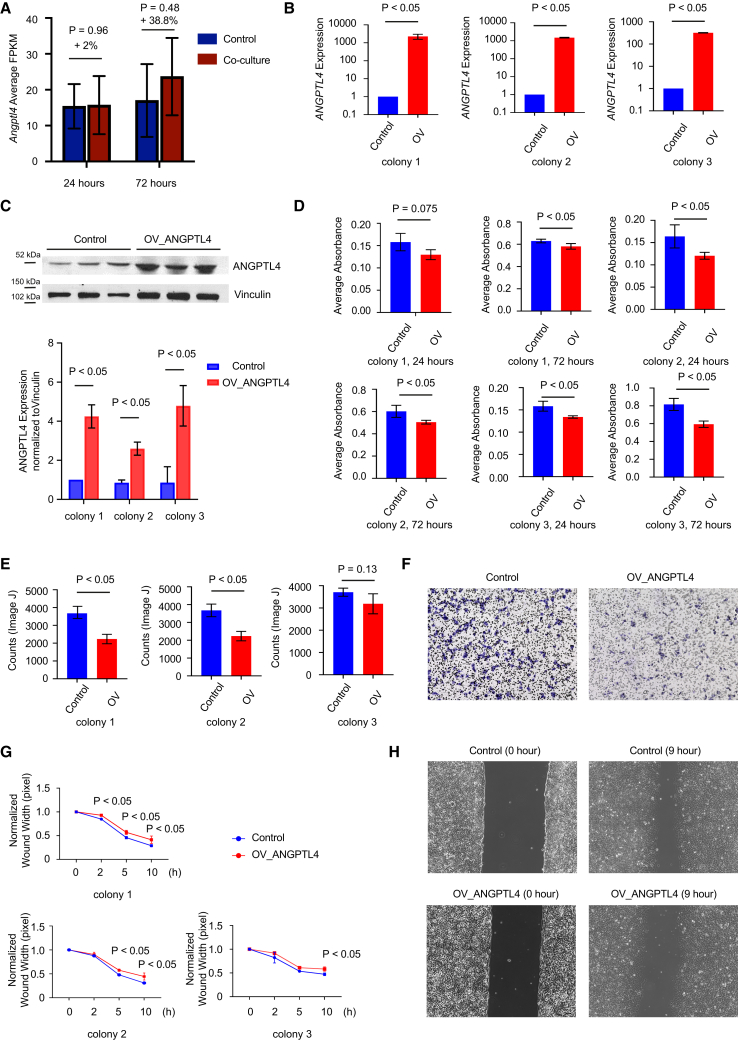
Over-expression of ANGPTL4 in fibroblasts reduces the proliferation and migration of fibroblasts (A) RNAseq conducted on fibroblast reveals higher expression of *Angptl4* when co-cultured with KPC cancer cells for 24 and 72 h. Data are represented as mean ± SD. (B) Expression of *ANGPTL4* in control and ANGPTL4-overexpressed fibroblasts (OV_ANGPTL4) determined by qPCR. Data are represented as mean ± SD. (C) Western blot analysis of ANGPTL4 and Vinculin (loading control) in control and OV_ANGPTL4 fibroblasts. Data are represented as mean ± SD. (D) MTT assay results comparing the proliferation of control and OV_ANGPTL4 (3 different colonies) fibroblasts. Cells were seeded, and incubated for 24 and 72 h, followed by MTT solution addition for 4 h. With absorbance measured at 570 nm. Data are represented as mean ± SD. (E) Migration abilities of control and OV_ANGPTL4 fibroblasts were assessed using *trans*-well assay, with quantitative evaluation performed using ImageJ software. Data are represented as mean ± SD. (F) Representative images of the *trans*-well migration assay for control and OV_ANGPTL4 fibroblasts. (G) Wound healing assay measuring the migration of control or OV_ANGPTL4 fibroblasts. Data are represented as mean ± SD. (H) Representative images from the wound healing assay at 0 and 9 h.

**Figure 10:**
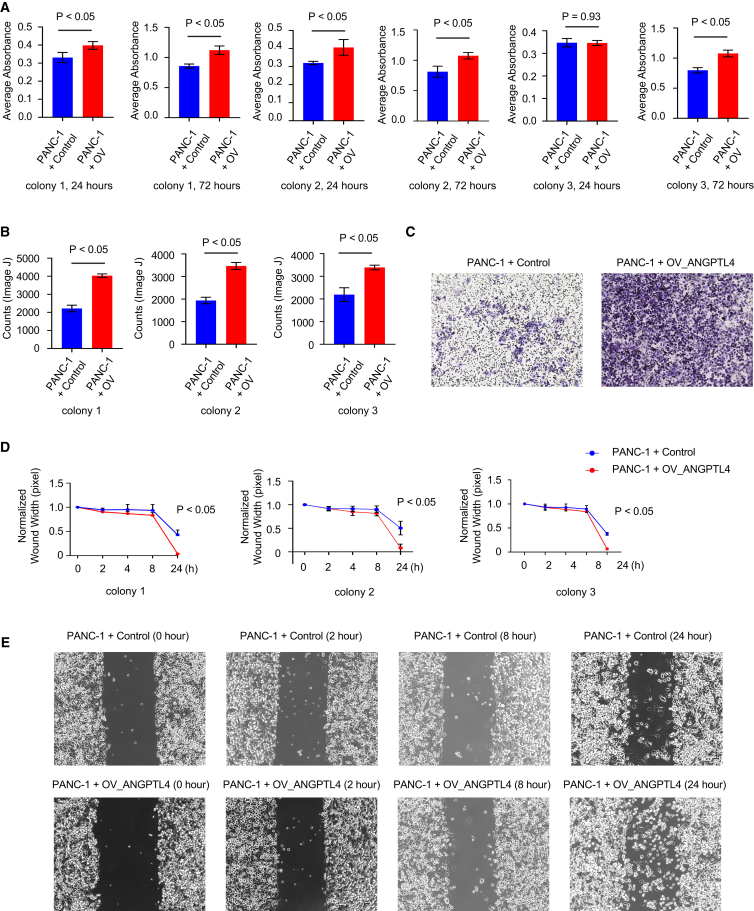
Over-expression of ANGPTL4 in fibroblasts induces pancreatic cancer cell proliferation and migration in a paracrine manner (A) MTT assay for PANC-1 cells incubated with conditioned culture media from control or OV_ANGPTL4 (3 different colonies) fibroblasts. PANC-1 cells were seeded and after 24 and 72 h of incubation, MTT solution was added and incubated for 4 h. Absorbance was measured at 570 nm. Data are represented as mean ± SD. (B) The migration abilities of PANC-1 cells to control and OV_ANGPTL4 fibroblasts were measured by *trans*-well assay. The quantitative evaluation was conducted by ImageJ software. Data are represented as mean ± SD. (C) Representative migration assay panels of PANC-1 cells co-cultured either with control or OV_ANGPTL4 fibroblasts. (D) The migration of PANC-1 cells with conditioned culture media from control or OV_ANGPTL4 fibroblasts was measured by wound healing assay. Data are represented as mean ± SD. (E) Representative panels of PANC-1 cells incubated with conditioned culture media from control or OV_ANGPTL4 fibroblasts wound healing assay (0, 2, 8 and 9 h).

Throughout the time course, paracrine factors may be produced that stimulate *Angptl4* expression in fibroblasts. Expression of *Angptl4* in wild-type fibroblasts was 38.8% elevated 72 h after co-culture with primary KPC mouse pancreatic cancer cells compared to the expression without co-culture with KPC cancer cells (Figure 9A), suggesting that cancer cell-driven paracrine factors may contribute to regulating *Angptl4* expression. To further investigate the roles of ANGPTL4 in fibroblasts and cancer cells, we generated a CRISPR-based *ANGPTL4*-overexpressing SV-80 fibroblast cell line (referred to as OV_ANGPTL4). Over-expression of ANGPTL4 was confirmed at both RNA and protein levels (Figures 9B and 9C). Interestingly, ANGPTL4 over-expression in fibroblasts led to a reduction in fibroblast proliferation (Figure 9D), migration (Figures 9E and 9F), and would healing capacities (Figures 9G and 9H). However, when we applied conditional medium from OV_ANGPTL4 fibroblasts to PANC-1 pancreatic cancer cells, we observed a significant increase in cancer cell proliferation (Figure 10A), migration (Figures 10B and 10C), and would healing (Figures 10D and 10E), compared to medium from control fibroblasts.

In summary, over-expression of ANGPTL4 in fibroblasts promotes pancreatic cancer cell proliferation, migration, and would healing through paracrine mechanism, but does not enhance these processes in fibroblasts themselves via an autocrine pathway. Given that cancer cell-driven paracrine signals appear to regulate *Angptl4* expression in fibroblasts, ANGPTL4 may act as a reciprocal factor, creating a feedback loop that supports pancreatic cancer progression.

### Expression of *Lama2* in fibroblasts is decreased in human and murine pancreatic cancer

**Figure 11:**
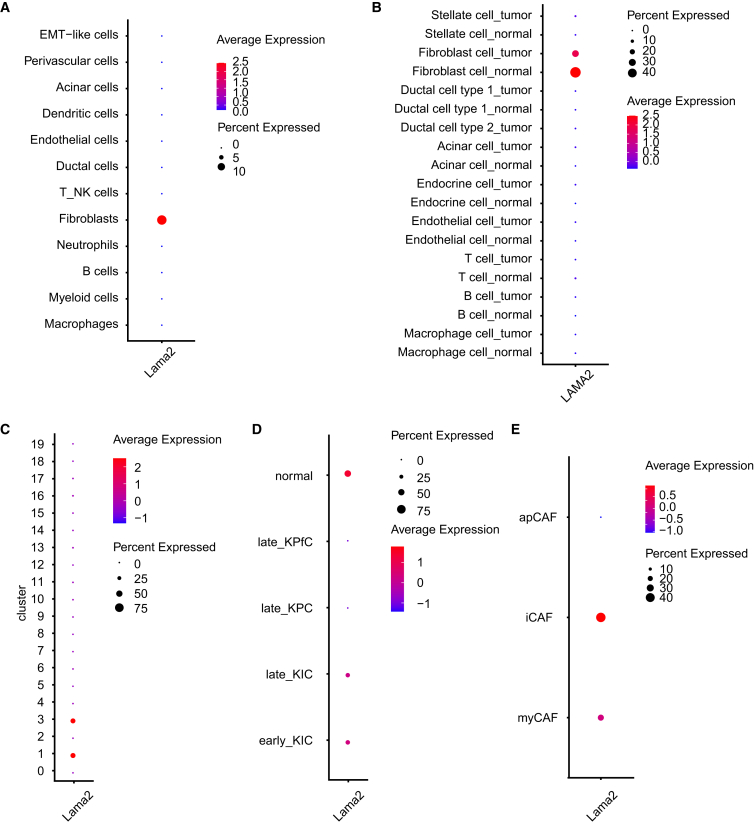
Expression of *Lama2* in fibroblasts is decreased in human and murine pancreatic cancer from scRNA-seq analyses (A) *Lama2* expression in different cell types in KPC mice based on scRNA-seq data from Elyada et al. (B) Analysis with human scRNA-seq data from Peng et al. shows fibroblast-dominated and reduced *LAMA2* expression in tumor. (C) Expression of *Lama2* in 19 different clusters. UMAP plot based on scRNA-seq data from Hosein et al. for distinct cell populations and 19 clusters are shown in Figures 6B and 6C. (D) *Lama2* expression in fibroblast clusters (clusters 1, 3, and 13) in different pancreatic cancer mouse models in distinct time points (early: 40 days, late 60 days). (E) *Lama2* expression in CAF subtypes based on fibroblast scRNA-seq data from Elyada et al.

Among the 26 narrowed-down targets in our dataset, we also identified several genes associated with the ECM which were downregulated (Figure 6A). Single-cell RNA-sequence data from Elyada et al.^20^ revealed that *Lama2* expression is predominantly observed in fibroblasts from KPC mice (Figures 11A and S3), Further analysis of scRNA-seq data from human PDAC and healthy control samples (Peng et al.)^30^ also demonstrated that *LAMA2* expression is prominent in fibroblasts from healthy control samples, but reduced in fibroblasts from human PDAC (Figures 11B and S4). Notably, *Lama2* expression was observed in distinct fibroblast clusters compared to *Angptl4* expression. When analyzing *Lama2* expression in murine scRNA-seq data from Hosein et al.,^29^ we observed its presence in fibroblast clusters 1 and 3 (Figures 11C and S1), while *Angptl4* expression was dominant in cluster 13 (Figures 6E and S1), Although no clear differences in *Lama2* expression were detected between early and late stages in KIC fibroblasts, we observed a reduction in *Lama2* expression in fibroblasts from late-stage KPC and KPfC mice (Figures 11D and S2). Additionally, scRNA-seq data from Elyada et al.^20^ indicated that *Lama2* expression is primarily associated with iCAF subtype (Figures 11E and S5).

In summary, *Lama2* expression in fibroblasts is decreased in both human and murine pancreatic cancer models. Moreover, *Lama2* and *Angptl4* are predominantly expressed in distinct fibroblast subtypes.

### LAMA2 inhibits pancreatic cancer cell proliferation and migration

**Figure 12:**
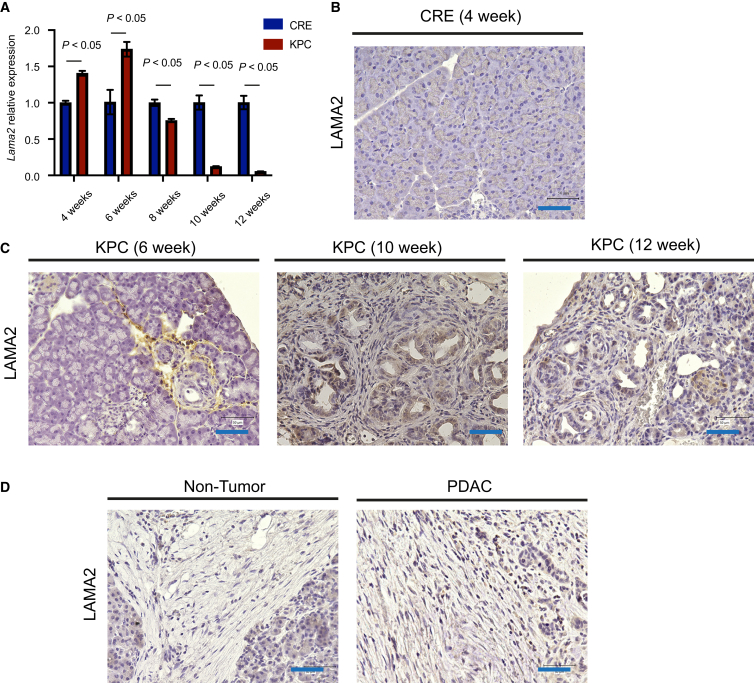
*Lama2* downregulation in KPC fibroblasts throughout the entire time course (A) RT-qPCR to validate the downward expression of *Lama2* in KPC-fibroblast as compared to CRE-fibroblast across time points. Data are represented as mean ± SD. (B) Immunohistochemistry conducted on CRE and (C) KPC murine pancreatic tissues to show stromal expression of LAMA2 (Bar: 50 μm). (D) Immunohistochemistry conducted on representative human pancreatic tissues (non-tumor and PDAC) to show very weak expression of LAMA2 both in non-tumor samples and in PDAC (Bar: 50 μm).

We observed time-course-dependent down-regulation of *Lama2* in KPC fibroblasts compared to its expression in CRE control fibroblasts (Figure 12A). Immunocytochemistry analysis revealed stromal expression of LAMA2 in 12-week-old KPC mice, although their expression was weak at 10 and 12 weeks, and absent in in CRE mice (Figures 12B and 12C). In human samples, LAMA2 expression was barely detectable in both non-tumor and PDAC specimens (Figure 12D). The lack of LAMA2 staining in CRE mice may be due to a lower number of fibroblasts, while in human samples, LAMA2 down-regulation may have already occurred at this stage. We also performed immunocytochemistry for FBLN2 and THBS1, noting strong stromal expression of FBLN2 in PDAC, whereas THBS1 was not predominantly expressed in fibroblasts (Figure S6).

**Figure 13:**
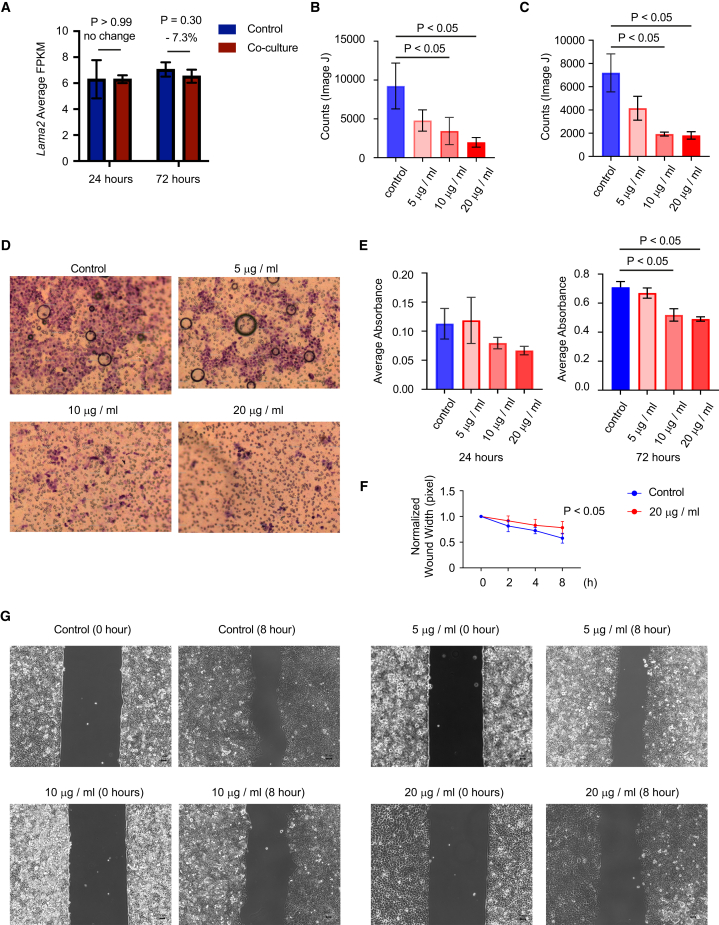
LAMA2 inhibits pancreatic cancer cell proliferation and migration (A) RNAseq conducted on fibroblast reveals lower expression of *Lama2* when co-cultured with KPC cancer cells for 72 h. Data are represented as mean ± SD. (B) The invasion by different LAMA2 recombinant protein concentrations was assessed by counting the number of KPC3595 cells that migrated through the ECM toward the LAMA2 in the lower chamber using ImageJ for quantification. Data are represented as mean ± SD. (C) The migration abilities of KPC3595 cell lines were measured by *trans*-well assay. The quantitative evaluation was conducted by ImageJ software. Data are represented as mean ± SD. (D) Representative panels of migration assay. (E) MTT assay for KPC3595 cells after incubation with different LAMA2 recombinant protein concentrations. Data are represented as mean ± SD. (F) The migration of KPC3595 was measured by wound healing assay with 20 μg/mL Lama2 recombinant protein. Data are represented as mean ± SD. (G) Representative panels of KPC3595 would healing assay (0 and 8 h).

In co-culture experiments, we found that *Lama2* expression in wild-type fibroblasts was 7.3% down-regulated 72 h after co-culture with KPC cancer cells, compared to the expression without co-culture with KPC cancer cells (Figure 13A). Given that baseline LAMA2 in wild-type fibroblasts was already low (Figure 12B), only a mild down-regulation effect was observed following co-culture. To further explore the functional effects of LAMA2, we used recombinant LAMA2 in murine pancreatic cancer cells (KPC3595). LAMA2 stimulation led to reduced cancer cell invasion (Figure 13B), migration (Figures 13C and 13D), proliferation (Figure 13E), and would healing (Figures 13F and 13G).

In summary, our study provides the comprehensive analysis of temporal transcriptional changes in fibroblasts during early-stage pancreatic carcinogenesis, highlighting the dynamics of fibroblast subtypes, including genes related to cell cycle regulation, adipogenesis, and ECM remodeling. ANGPTL4 may play a role in creating a feedback loop that supports pancreatic cancer progression, while the down-regulation of the ECM protein LAMA2 is likely associated with pancreatic cancer advancement.

## Discussion

A stroma-rich microenvironment is a defining feature of pancreatic cancer, and mounting evidence highlights the multifaceted impact of fibroblasts within this context. Fibroblasts, the predominant stromal component, wield influence over cancer progression, invasion, and metastasis,^31^ by modeling the extracellular matrix (ECM), fostering cancer cell survival, promoting migration, enabling immune evasion, and impeding drug delivery.^20^^,^^32^^,^^33^ It has been recognized that tumor associated fibroblasts exert diverse functions and do not solely act tumor suppressive or tumor promoting. Early studies have shown that targeting stromal α-SMA positive fibroblasts in PDAC mouse models through ablation may paradoxically support tumor development.^17^ Recently, targeting the stroma using a pan-lysyl oxidase inhibitor has been shown to reduce invasion and metastasis and chemotherapy efficacy in the KPC mouse model.^34^ Functional diversity in fibroblasts has been linked to various subtypes identified through advanced single-cell RNA sequencing and their diverse cellular origins^22^^,^^35^^,^^20^^.^. Pancreatic stellate cells, resident fibroblasts, and mesenchymal stem cells are major sources of cancer-associated fibroblasts. To identify whether cancer-associated fibroblasts across various time points in our KPC model are derived from pancreatic stellate cells or from other cell types, a lineage-tracing system would be required since we cannot identify cellular origin after (*trans*-) differentiation into cancer-associated fibroblasts.

In contrast to its role in cancer progression, the effects of stromal cells during early carcinogenesis has not been well elucidated. Pancreatic cancer is thought to develop from two distinct precursor lesions: intraductal papillary mucinous neoplasia (IPMN) and pancreatic intraepithelial neoplasia (PanIN).^36^ PanIN lesions are thought to develop from acinar cells via acinar-to-ductal metaplasia (ADM). For both precursor lesions, KRAS mutations seem to be the key driver.^37^^,^^38^ Remarkably, oncogenic KRAS has been demonstrated to activate fibroblasts even before ADM formation, establishing an immune-suppressive stroma in the early-stages of carcinogenesis.^39^ KRAS signaling in acinar cells and inflammatory pathways in immune cells could act as upstream factors activating and supporting (*trans*-) differentiation into cancer-associated fibroblasts in a paracrine manner. It is therefore possible that KRAS and inflammatory pathways are showing a lower expression trend in primary isolated fibroblasts. This highlights the pivotal role of fibroblasts not only in creating a tumor-promoting stromal microenvironment but also in driving early tumor development. Recent breakthroughs in single cells RNA sequencing have also unveiled a transformative shift in fibroblast subtypes, linked to the tumor’s evolution from early to late PDAC stages. This underscores the remarkable plasticity of fibroblasts during disease progression,^40^ prompting a need for in-depth functional exploration and shedding light on crucial roles in the early phases of PDAC development. By amalgamating literature on fibroblast relevance in early PDAC and recognizing the impact of the absence of early diagnostic markers on prognosis,^41^ our work focuses on molecular signaling pathways that govern their interaction with pre-neoplastic cells in early PDAC stages.

Our study is to dissect transcriptional dynamics in fibroblasts during early-stages of PDAC using the KPC mouse model. In the current study, we isolated primary fibroblasts in KPC mice at different time points during carcinogenesis, i.e., during ADM and PanIN formation, as precursor lesions of pancreatic cancer. Here we show that during the early phase of tumorigenesis, only a few significant changes in gene expression were evident in KPC-fibroblasts versus control CRE-fibroblasts. Most changes were observed at week 12, when KPC mice are expected to harbor high-grade PanIN lesions.^25^ As a relevant pancreatic disease mouse model, Hingorani et al.^38^ have established KC (*Ptf1a/p48-Cre; lox-stop-lox-Kras*^*G12D/+*^ and *Pdx-1-Cre; lox-stop-lox-Kras*^*G12D/+*^) mouse models. Endogenous *Kras*^*G12D/+*^ expression in KC mice also induces PanINs but to a slower extent compared to the KPC mice. It is therefore possible that gene expression dynamics in fibroblasts could be slowed down in KC fibroblasts in comparison to KPC fibroblasts. Further, p53R172H mutation can activate additional signaling in KPC mice. Several pathways were enriched in KPC-fibroblasts during carcinogenesis such as E2F targets, G2M checkpoints, and Myc targets that exhibited a continuous upward trend across all time points, suggesting increased proliferation of these cells during early carcinogenesis. This fits well with the observation of enhanced desmoplasia already in PanIN lesions.^42^^,^^43^ Interestingly, an upward trend, especially at the 12-week time-point was seen for adipogenesis, reactive oxygen species, and fatty acid metabolism, suggesting changes in the metabolism of these cells during carcinogenesis. Next, linear regression analysis was carried out and genes were selected that were either secreted or located on the cell surface, making them potential candidates for mediating paracrine effects within the tumor microenvironment, including effects on pre-neoplastic cells. This analysis revealed 96 genes, which exhibited continuous increase or decrease of throughout the entire time course. Among the 96 identified genes, 26 genes were also identified as relevant DEGs at 12-week time point, which is critical time point as it marks transition from pre-neoplastic lesions to invasive stages.^25^ We identified *Angptl4* as a significantly upregulated gene that belonged to the adipogenesis hallmark set which was the pathway positively enriched across the time points of our GSEA analysis.

ANGPTL4, a secreted protein within the angiopoietin-like factors superfamily, plays multifaceted roles such as mediating angiogenesis, cell differentiation, glucose regulation, tumorigenesis, lipid metabolism by inhibiting lipoprotein lipases, and redox control.^44^ Its transcriptional control is subject to modulation by multiple factors, including PPARα, PPARβ/δ, PPARγ, TGF-β in conjunction with PPARs^45^ signaling, HIF-1α, and nutritional/hormonal cues.^46^^,^^47^ The tumor-promoting role of ANGPTL4 has been described in several cancer types, for instance, increase proliferation and promote metastasis in gastric^48^ and hepatic cell carcinoma,^49^ increase cell migration and invasion in colorectal cancer,^50^ promote tumor progression in oral cancer^51^ and promote pro-angiogenic factors in Kaposi-sarcoma.^52^^,^^53^ Further, ANGPTL4 in KRAS^G12D^-expressing pancreatic epithelial cells enhances ADM and PanIN formation,^54^ a tumor-promoting role of ANGPTL4 is suggested in a cell-autonomous manner. In connection to fibroblast, prior investigation revealed elevated Angptl4 expression in CAFs of breast,^55^ gallbladder,^56^ and gastric cancer.^57^ However, in context to PDAC, we show the elevation of *Angptl4* in KPC-fibroblasts during carcinogenesis (Figure 6A). It is tempting to speculate that oncogenic KRAS^G12D^-expressing pancreatic acinar cells secrete paracrine factors that activate signaling pathways in fibroblasts to induce *Angptl4* expression.^54^ Our data from co-culture experiments of KPC fibroblasts with primary pancreatic cancer cells also showed an increase in *Angptl4* expression compared to KPC fibroblasts without co-culture (Figure 9A). The overexpression of ANGPTL4 in fibroblasts induces pancreatic cancer cell proliferation, migration, and wound healing in paracrine manner, while not exhibiting autocrine effects within the fibroblasts themselves. This suggests that factors secreted by cancer cells may play a crucial role in modulating ANGPTL4 expression in fibroblasts, indicating that ANGPTL4 could function as a reciprocal factor that contributes to a feedback loop promoting pancreatic cancer progression. Additionally, our findings suggest that the proportion of pancreatic CAF subtypes can vary dynamically over time in different mouse models. Notably, the expression of *Angptl4* in fibroblasts correlates more closely with the expression of markers associated with apCAFs. While there is a mounting body of evidence linking elevated expression of ANGPTL4 to various malignancies, its specific role in cancer metabolism is less clear, except for few studies in non-small cell lung carcinoma, where it has been demonstrated to promote energy metabolism through increased utilization of glutamine and promote fatty acid metabolism.^58^ Furthermore, ANGPTL4 has been observed to enhance the pro-survival intracellular oxygen to H_2_O_2_ ratio, leading to anoikis (anchorage independent growth of cells-feature of cancer cells) resistance in tumors and promoting tumor-growth via the PI3K/PKBα/ERK signaling pathway.^59^ Our findings of the upregulation of hallmark sets related to adipogenesis, fatty acid metabolism, and the ROS pathway in 12-week KPC-fibroblasts, suggest that increased expression of *Angptl4* by fibroblasts in KPC mice may contribute to metabolic reprogramming and redox regulation during the early-stage of pancreatic carcinogenesis. The upregulation of hallmark sets related to adipogenesis and fatty acid metabolism observed in 12-week KPC fibroblasts - following the inflammatory and fibrotic stages – remains unclear. One possibility is that specific CAF subtypes exhibit distinct metabolic activities, highlighting the need for further investigation into CAF-subtype-dependent metabolic regulation. Additionally, future pharmacological studies focusing on ANGPTL4 inhibitors should be prioritized in pancreatic disease research. A monoclonal antibody, mAb 14D12, has been developed to target a specific epitope (Gln29-His53) on ANGPTL4, effectively inhibiting its function.^60^ ANGPTL4 inhibitors show promise for treating lipid disorders associated with metabolic syndrome, type 2 diabetes, and familial hyperlipidemia, with preclinical development currently underway. Recent works have shown that over-expression of ANGPTL4 is linked to the down-regulation of the mRNA levels of ECM-related genes in triple-negative breast cancer cell lines.^61^ Hence, it may be possible that ANGPTL4 plays a role in downregulating ECM-related genes in pancreatic cancer. Since ANGPTL4 undergoes post-translational modifications leading to multiple functional domains (fANGPTL4, nANGPTL4, cANGPTL4),^62^ future investigations need to be centered on protein levels to elucidate the diverse effects of ANGPTL4.^47^

The dynamics of the extracellular matrix (ECM) within the tumor microenvironment are pivotal in driving early metastasis and drug resistance in PDAC progression,^4^ in which fibroblasts play a crucial role.^63^ Growing evidence show that fibroblast mediated ECM-degradation might be necessary to pave the way for cancer cells to modify, proliferate and undergo metastatic invasions^64^ via a set of matrix metalloproteinases (MMPs) and downregulation of Dyn2, but the mechanisms underlying the fibroblast mediated ECM degradation largely remain unknown.^65^ Specifically, MMP-2, -3, -9, and -14 are often upregulated and linked to ECM remodeling in multiple cancer types.^66^ During tumor progression, MMP-2/9/14 facilitate tumor cell invasion by breaking down collagen IV in the basement membrane, leading to metastasis.^67^^,^^68^ In our 12-week DEG analysis, we observed the upregulation of MMP-9 and MMP-14, along with the consistent downregulation of *Col12a1* across all time points. Recent findings indicate that the lysyl oxidase family (Loxl1-Loxl4), which enhances collagen crosslinking, may offer the potential for stroma-focused therapies.^34^ Notably, *Loxl2* was found downregulated in KPC fibroblasts during carcinogenesis, suggesting Loxls' role in metastatic invasion in PDAC.^64^ However, given that fibroblast-related stromal complexities in cancers, further research is required to determine whether early PDAC stroma is tumor-suppressive or promotes tumors.^22^ Among the 26 narrowed-down targets in our analysis, we observed that 10 genes associated with the ECM were downregulated. These findings collectively suggest potential ECM degradation, implying a risk of metastasis invasion in PDAC progression beyond the 12-week mark. In this regard, we also validated the expression of *Lama2* the most significantly downregulated gene at 12 weeks by RT-qPCR in cells and tissues from CRE and KPC mice at all time points. Interestingly, it has been shown that the down-regulation of LAMA2 promoted migration and invasion in lung adenocarcinoma.^69^ Consequently, exploring the paracrine role of fibroblast mediated LAMA2 on pre-neoplastic cells presents an intriguing avenue for future studies. Our data from co-culture experiments of pancreatic fibroblasts with primary pancreatic cancer cells did not show a significant difference but a slight down-regulation in *Lama2* expression. We will further investigate to clarify why pancreatic fibroblasts did not show any significant downregulation of *Lama2* after co-culture with pancreatic cancer cells.

Our study has some limitations. In an *in vitro* setting, the culture of fibroblasts may result in the loss of certain microenvironment effects, such as communication with epithelial cells, cancer cells, and immune cells. It is therefore challenging to identify paracrine or reciprocal factors playing roles in pancreatic carcinogenesis. So far, there is rare single-nucleus RNA sequencing data available for elucidating the role of fibroblasts in early-stage pancreatic carcinogenesis. To that end, it may be interesting to analyze the early-stage pancreatic carcinogenesis directly without cell culture by quickly freezing the samples and performing snRNA-seq in the future. Identification of specific fibroblast subtypes and markers during early stages of pancreatic carcinogenesis has challenged further studies.

In summary, our study sheds light on the transcriptional dynamics of fibroblasts in early pancreatic carcinogenesis and sets the stage for future investigations into the mechanisms driving fibroblast-mediated expression of *Angptl4* and ECM genes, along with their potential roles in crosstalk with pre-neoplastic cells during early cancer development.

### Limitations of the study

In an *in vitro* setting, the culture of fibroblasts may result in the loss of certain microenvironment effects, such as communication with epithelial cells, cancer cells, and immune cells. It is therefore challenging to identify paracrine or reciprocal factors playing roles in pancreatic carcinogenesis in general. So far, there is rare single-nucleus RNA sequencing data available for elucidating the role of fibroblasts in early-stage of pancreatic carcinogenesis. To that end, it may be interesting to analyze the early-stage pancreatic carcinogenesis directly without cell culture by quickly freezing the samples and performing snRNA-seq in the future. Further identification of specific fibroblast subtypes and markers during early-stage of pancreatic carcinogenesis has challenged further studies.

## Resource availability

### Lead contact

Further information and requests for resources and reagents should be directed to and will be fulfilled by the lead contact, Yoshiaki Sunami (yoshiaki.sunami@uk-halle.de).

### Materials availability

This study did not generate new unique reagents.

### Data and code availability

• RNA sequencing data have been deposited at NCBI GEO: GSE270121 and are publicly available as of the date of publication. All accession numbers used for this study are listed in the key resources table. Original western blot images have been deposited at the Sachsen-Anhalt Data Archive and Repository (SADAR) of the Martin-Luther-University Halle-Wittenberg and are publicly available (https://doi.org/10.25673/1914118-8).
• This article does not report original code.
• Any additional information required to reanalyze the data reported in this article is available from the lead contact upon request.

## Acknowledgments

We greatly thank Dr. Ela Elyada, Dr. Lindsey Baker, and her colleagues for a fibroblast-enriched sc-RNAseq dataset as CellRanger output files along with cell type annotations. We also thank Dr. Bo Kong for the murine pancreatic cancer cell lines. The work was conducted within the Research Training Group 2751 (InCuPanc), funded by the 10.13039/501100001659Deutsche Forschungsgemeinschaft (DFG, German Research Foundation) 449501615 (to JK and MG).

## Author contributions

Conceptualization: Y.S. and J.K.; methodology: N.O., J.H., N.J., D.V., A.Z., B.T., and M.G.; validation: N.O., M.H., I.E., M.G., Y.S., and J.K.; formal analysis: N.O., M.G., and Y.S.; investigation: N.O., J.H., N.J., D.V., A.Z., and M.G.; writing - original draft: N.O., M.G., and Y.S.; writing - review and editing: M.G., Y.S., and J.K.; visualization: N.O., M.G., and Y.S.; funding acquisition: M.G. and J.K. All authors reviewed and approved the article.

## Declaration of interests

The authors declare no competing or financial interests.

## STAR★Methods

### Key resources table

**Table:** 

REAGENT or RESOURCE	SOURCE	IDENTIFIER
**Antibodies**		

**α**-SMA	Abcam	Cat# ab5694; RRID: AB_2223021
ANGPTL4	Invitrogen	Cat# PA5-26216; RRID: AB_2543716
CK19	Invitrogen	Cat# MA5-31977; RRID: AB_2809271
FAP	Gentex	Cat# GTX35261
Fibulin 2	Invitrogen	Cat# PA5-51665; RRID: AB_2641632
LAMA2	BIOSS	Cat# bs-8561R
Vinculin	Cell signaling tech.	Cat# 4650; RRID: AB_10559207
Goat anti rabbit	Invitrogen	Cat# G21234; RRID: AB_2536530

**Biological samples**		

Human subjects	This paper	N/A
Tissue microarrays	Haeberle et al.^70^	N/A

**Chemicals, peptides, and recombinant proteins**		

High glucose DMEM	Sigma-Aldrich	D5796
Neo-Clear™ Xylene	Sigma-Aldrich	109843 5000
FBS	Bio&Sell	S0615HI
Mayer’s-Hematoxylin solution	Sigma-Aldrich	109249 0500
Eosin Y-solution 1%	Carl Roth	3137.1
Absolute Ethanol	Carl Roth	K928.4
TRIS	Carl Roth	AE15.1
EDTA	Sigma-Aldrich	E1644-250G
Citric-Acid 1-hydrate	AppliChem	141018.1211
Sodium Citrate Trisodium salt, Dihydrate	Sigma-Aldrich	S-4641
PBS (Phosphate buffered saline)	AppliChem	A0964,9050
BSA (Bovine Serum Albumin)	Sigma-Aldrich	A3912-100G
Tween 20	SERVA	37470.01
Xylene	Carl Roth	9713.1
Entellan	Sigma-Aldrich	107961 0100
Envision+System-HRP	DAKO	K4003
Antibody Diluent	DAKO	K8006
DAB+ Chromogen	DAKO	K3468
HCl	Sigma-Aldrich	100316 1000
NaOH	Carl Roth	6771.3
2-Propanol	Merck	109634 1011
Lipofectamine 3000	Thermo Fischer Scientific	L3000001
Puromycine	Gibco	A11138-02
PVDF	Merck	IPVH00010
MTT	Sigma-Aldrich	M2128-1G
DMSO	Carl Roth	A994.2
Recombinant LAMA2	USbiological Life Science	155597

**Critical commercial assays**		

Alcian Blue Staining Kit	Sigma-Aldrich	132657 0001
Direct-zol RNA MiniPrep	Zymo Research	R2052
Ibidi-culture inserts 2-well chambers for wound healing assay	Ibidi	81176
Picro-Sirius Red Staining Kit for Collagen I & III	Morphisto	13425.00250
24-well wells with 8 **μ**m pore inserts for migration assay	Greiner bio-one	662638

**Deposited data**		

RNA sequencing data	This paper	NCBI GEO: GSE270121
Western Blot data	This paper	https://doi.org/10.25673/1914118-8
The pancreas scRNA-seq dataset from the Tabula Muris project	Tabula Muris Consortium et al. Nature^71^	N/A
Mouse pancreatic cancer scRNA-seq	Elyada et al.^20^	Dr. Lindsey Baker
Mouse pancreatic cancer scRNA-seq	Hosein et al.^29^	NCBI GEO: GSE125588
Human scRNA-seq data	Peng et al.^30^	NGDC BioProject: PRJCA001063

**Experimental models: Cell lines**		

SV-80	Cellosaurus	RRID: CVCL_0541
Primary mouse KPC pancreatic cancer cells	Kong et al.^72^	N/A
Primary mouse KPC3595 pancreatic cancer cells	Benitz et al.^73^	N/A

**Experimental models: Organisms/strains**		

Mouse: *Ptf1a-Cre*	The Jackson Laboratory	RRID: IMSR_JAX: 019378
Mouse: *LSL-KrasG12D*	The Jackson Laboratory	RRID: IMSR_JAX: 008179
Mouse: *LSL-Trp53R172H*	The Jackson Laboratory	RRID: IMSR_JAX: 008652

**Oligonucleotides**		

Primer: *Angptl4* (mouse) S: 5'-GAT GGC TCA GTG GAC TTC AAC C-3' AS: 5'-TGC TAT GCA CCT TCT CCA GAC C-3'	Metabion International AG	N/A
Primer: *Lama2* (mouse) S: 5'-TGG AAG TAG CCG AAC CAG GAC A-3' AS: 5'-CAC CTG GTT CAG AAC GAA GTC G-3'	Metabion International AG	N/A
Primer: ***β****-actin* (mouse) S: 5’-CCC ATC TAC GAG GGC TAT GC-3’ AS: 5’-ATG TCA CGC ACG ATT TCC CT-3’	Metabion International AG	N/A
Primer: *B2M* (human) S: 5’-GGC TAT CCA GCG TAC TCC AA-3’ AS: 5’-GTC GGA TGG ATG AAA CCC AGA-3’	Metabion International AG	N/A
Primer: *YWHAZ* (human) S: 5’-AGC AGG CTG AGC GAT ATG AT-3’ AS: 5’-TCT CAG CAC CTT CCG TCT TT-3’	Metabion International AG	N/A

**Recombinant DNA**		

ANGPTL4 CRISPR activation plasmid	SantaCruz biotechnology inc.	sc-401369-ACT
Control CRISPR activation Plasmid	SantaCruz biotechnology inc.	sc-437275

**Software and algorithms**		

GraphPad Prism v8.4	GraphPad	N/A
R-package edgeR	Robinson et al.^74^	https://bioconductor.org/packages/release/bioc/html/edgeR.html
R-package clusterProfiler (v.6.2)	Guangchuang Yu et al.^75^	https://bioconductor.org/packages/release/bioc/html/clusterProfiler.html
CIBERSORTx	Newman et al.^76^	https://cibersortx.stanford.edu/
R-package Seurat v4.3.0	Hao et al.^77^	https://satijalab.org/seurat/
R-package Seurat v5.1.0	Hao et al.^77^	https://satijalab.org/seurat/
R-package scDblFinder v1.18.0	Germain et al.^78^	https://bioconductor.org/packages/release/bioc/html/scDblFinder.html

### Experimental model and study participant details

#### Mouse models

To investigate the role of early pancreatic carcinogenesis, the KPC mouse model expressing *KRAS* and *Trp53* mutations under the *Ptf1a* promotor^24^ (*Ptf1a/p48-Cre; lox-stop-lox-Kras*^*G12D/+*^*; lox-stop-lox-Trp53*^*R172H/+*^) was used. Acinar cell specific expression of oncogenic *Kras* and *Trp53* mutation has been shown to promote invasive pancreatic adenocarcinoma within 14-16 weeks. These mice show a clinical disease that is comparable to the human disease.^24^^,^^25^^,^^79^ CRE-mice (*Ptf1a-Cre*) were analyzed as a control group without the development of any pancreatic cancer or its precursors. The genetic backbone of the mouse model was C57BL/6J. Breeding and generation of the mice used for the study were approved by the state administration office (Landesverwaltungsamt) Sachsen-Anhalt (registration number MLU 2-1348).

#### Isolation and culture of primary pancreatic fibroblasts

Pancreatic fibroblasts were isolated from pancreata of 4, 6, 8, 10, and 12-week-old KPC- and CRE-mice as previously described with a few modifications.^80^ The tissue was removed from animals and quickly stored in HBSS on ice. The organs were minced with a sterile scalpel and digested at 37°C for 30 minutes in an enzyme buffer consisting of GBSS with salt, 0.02% Pronase (Roche), 0.05% Collagenase P (Roche) and 0.1% DNAse (Roche). The digested pancreata were filtered through a 100 μm cell strainer (Corning) which was flushed with HBSS to increase the yield. The mixture was centrifuged at 450 g for six minutes at 4°C. After a washing step with GBSS with salt, the pellet was re-suspended in 4.75 ml GBSS with 0.3% bovine serum albumin (BSA) (Sigma). 4 ml of Nycodenz-Solution (1.435 g Nycodenz by Alere Tech AS in 5 ml GBSS without salt) were layered carefully on top of this suspension, followed by 3 ml of GBSS with 0.3% BSA. The gradient was centrifuged for 20 minutes at 1400 g at 4°C. The cells in the layer just above the interface between the Nycodenz and GBSS were harvested and washed in GBSS with salt. The cells were re-suspended in culture medium (DMEM supplemented with 15% FBS, 1% penicillin-streptomycin, and 1% Amphotericin B) and transferred into a 6-well plate. Fibroblasts were cultured at 37°C with medium exchange 24 hours after cell isolation and subsequently every three days. Only primary fibroblasts passage 4 were used in this study. Sub-confluent fibroblasts were passaged with Trypsin-EDTA for three minutes and split into new dishes at a 1∶2 dilution.

#### Isolation and culture of primary pancreatic cancer cells

Primary pancreatic cancer cell line, generated from a KPC (*Ptf1a/p48-Cre; lox-stop-lox-Kras*^*G12D/+*^*; lox-stop-lox-Trp53*^*R172H/+*^) mouse, was kindly provided from Dr. Bo Kong (University of Heidelberg). The isolation protocol was previously described.^72^ KPC cancer cells were cultured in high glucose DMEM (D5796, Sigma-Aldrich) supplemented with 10% FBS (company and catalog number) and 1% penicillin/streptomycin (company and catalog number). For experiments with LAMA2 recombinant protein, we obtained KPC3595 pancreatic cancer cells, also kindly provided from kindly provided from Dr. Bo Kong (University of Heidelberg). KPC3595 cells were generated from KPC (*Ptf1a/p48-Cre; lox-stop-lox-Kras*^*G12D/+*^*; Trp53*^*lox/+*^) mouse, which has been previously described.^73^

#### Human subjects

Human pancreatic cancer samples were obtained from Martin-Luther-University Halle-Wittenberg, University Medical Center Halle, Germany. The study on human material was approved by the institutional ethics board of the medical faculty of the Martin-Luther-University Halle-Wittenberg and designated in accordance with the Declaration of Helsinki (approval number 2019-037). Written consent was obtained from all patients.

#### Tissue microarray

Tissue microarrays (TMAs) have been generated as previously described.^70^ The use of human samples was approved by the ethics committee at the University Hospital of Düsseldorf, Germany (study number: 5397). Scoring of staining intensity (0: none, 1: weak, 2: moderate, 3: strong) was manually conducted by two authors (N.O. and N.J.) independently. In case of inconsistency, third author (Y.S.) evaluated the staining intensity.

### Method details

#### Fibroblast Co-culture assay

Fibroblasts isolated from C57BL/6 wild-type mice were seeded onto the membrane of insert with 1 μm pore size and KPC cells were seeded on the 6-well plate. We used the same number of fibroblasts and KPC cells. Fibroblasts were cultured in fibroblast culture medium and KPC cells were cultured separately. Once the cells reached confluency, i.e., after 24 hours, the membrane inserts containing fibroblasts were put onto the 6-wells with KPC cells. After starting co-culture, we used DMEM medium for both the fibroblasts and KPC cells. RNA was isolated from co-cultured fibroblasts 24 and 72 hours after co-culture. Fibroblasts without co-culture with KPC cells were used as control.

#### Hematoxylin and Eosin staining

To evaluate the progression of pre-neoplastic lesions in pancreatic tissue sections from KPC and CRE mice spanning 4 to 12 weeks, Hematoxylin and Eosin staining were performed. Sections were de-paraffinized using Neo-Clear™ Xylene Substitute twice for 10 minutes followed by rehydration through a series of ethanol dilutions (100%, 50%, 25%), followed by brief washing step using monodest water. For hematoxylin staining, the sections were immersed in hematoxylin for 3 minutes, followed by a rinsing in deionized water. The stain was allowed to develop in tap water for 5 minutes. Further, eosin staining involves a 30-second immersion. Subsequently, the slides were dehydrated by placing them in 95% ethanol for three 5-minute intervals, followed by three 5-minute intervals in 100% ethanol, ensuring the removal of excess ethanol before transitioning to xylene for three 15-minute periods. Finally, the slides were covered using mounting medium.

#### Sirius red staining

The tissue sections from the mice pancreas were de-paraffinized in xylene twice for 10 minutes each, followed by rehydration washes in 100% ethanol for 2 minutes each. The sections were then sequentially washed in 95% ethanol for 2 minutes, 80% ethanol for 2 minutes, and 70% ethanol for 1 minute. Further, the sections were incubated in hematoxylin for 15 minutes and washed under running water for 8 minutes. Samples were incubated in Picrosirius red solution for 60 minutes according to the manufacturer’s guidance, followed by two washes in acidified water for 1 minute each, then sequentially washed in 70%, 80%, 95%, and 100% ethanol for 1 minute each. The sections were washed twice in xylene for 3 minutes each and mounted with a drop of the mounting medium before carefully placing a cover glass to avoid bubbles.

#### Alcian blue staining

Slides were deparaffinized and hydrated to distilled water, followed by staining in alcian blue solution (pH = 2.5) for 10 minutes according to manufacturer’s guidance. After washing in running tap water for 2 minutes and rinsing in distilled water, the slides were counterstained with reagent 2 (nuclear fast red albumin sulphate solution 0.1%) for 5 minutes. A 1-minute wash in running tap water was followed by dehydration through 95% alcohol and two changes of absolute alcohol, each for 3 minutes. The slides were then cleared in xylene and mounted using a mounting medium.

#### Immunohistochemistry

Glass slides were used to mount 4-μm-thick formalin-fixed paraffin-embedded pancreatic tissue sections from KPC and CRE mice, sampled at time points ranging from 4 to 12 weeks. These sections were subjected to staining with antibodies targeting ANGPTL4 and LAMA2. The tissue sections were first de-paraffinized using Neo-Clear™ Xylene Substitute twice, each time for 10 minutes. Subsequently, they were rehydrated through a series of ethanol dilutions (100%, 50%, 25%) and rinsed in monodest water. Heat-mediated antigen retrieval for both targets was accomplished using citrate buffer at a pH of 6.0. Following cooling in phosphate-buffered saline (PBS) with a pH of 7.4, sections were blocked at room temperature to prevent non-specific binding. This blocking process involved two steps: first, a 10-minute enzyme block, and then a 1-hour block with 1% BSA. Further, sections were incubated overnight in a humidified chamber at 4°C with 100 **μ**l primary antibody using a pre-optimized dilution (ANGPTL4: 1:50, LAMA2: 1:100, FBLN2: 1:500, CK19: 1:500, **α**-SMA: 1:125). Next, slides were washed three times in PBS/T for 15 minutes and incubated for 45 minutes at room temperature with a secondary antibody, DAKO Envision +System-HRP Labelled Polymer, followed by an additional washing step. Staining was visualized through incubation with 3,3-diaminobenzidine tetrahydrochloride solution (Dako DAB chromogen kit 1:400 dilution) for 3-4 minutes at room temperature. Sections were then counterstained with Mayer’s hematoxylin solution (Sigma-Aldrich, Saint Louis, MO, USA) and further the slides were mounted with Entellan ™ mounting media (Sigma-Aldrich, Saint Louis, MO, USA).

#### RNA isolation

Total RNA was extracted from KPC-fibroblasts and CRE-fibroblasts obtained from mice aged 4 to 12-weeks. RNA isolation was performed using the Direct-zol RNA Microprep Kit (Zymo Research). Briefly, the cells were homogenized in TRI Reagent using either a homogenizer or a sonicator. A Zymo-Spin IC column was used to collect 200 μl of the homogenate in a Collection Tube, followed by centrifugation for 30 seconds at 12,000 g. The column was then subjected to centrifugation for 30 seconds at 12,000 g after adding 400 μl of RNA pre-wash buffer and 700 μl of RNA wash buffer successively. To dry the column, it was transferred to a new collection tube and centrifuged for 3 minutes at 12,000 g. Next, the column was incubated with 10 μl of DNase/RNase-free water at room temperature for 5 minutes before centrifugation for 30 seconds at 12,000 g to elute the RNA. The eluted RNA was stored at -80°C for RNA-sequencing and qPCRs. The concentration of cellular/tissue RNA was determined using a Nanodrop Spectrophotometer from Thermo Scientific. The qPCR reactions were performed on a Quant studio3 in triplicates. Alternatively, for the amplification of RNA, 200 ng of total RNA was used as input as directed by RT-PCR and was performed using primers according to the manufacturer’s instructions. The mRNA expression was normalized to *B2M* and *YWHAZ* for human, and β-actin for mouse. RT-PCR experiments were performed using forward and reverse primers to quantify *ANGPTL4* (Human), *Angptl4* (mouse), *Lama2*, *B2M*, *YWHAZ*, and β-actin mRNA.

#### RNA-sequencing and gene set enrichment analysis

After RNA extraction and quality control, RNA samples were analyzed together with Novogene Co Ltd. (Cambridge, UK). A total amount of 1 μg RNA per sample was used as input material for the RNA sample preparations. Sequencing libraries were generated by Novogene using NEBNext® UltraTM RNA Library Prep Kit for Illumina® (NEB, USA) following manufacturer’s recommendations and index codes were added to attribute sequences to each sample. Briefly, mRNA was purified from total RNA using poly-T oligo-attached magnetic beads. Fragmentation was carried out using divalent cations under elevated temperature in NEBNext First Strand Synthesis Reaction Buffer (5X). First strand cDNA was synthesized using random hexamer primer and M-MuLV Reverse Transcriptase (RNase H-). Second strand cDNA synthesis was subsequently performed using DNA Polymerase I and RNase H. Remaining overhangs were converted into blunt ends via exonuclease/polymerase activities. After adenylation of 3’ ends of DNA fragments, NEBNext Adaptor with hairpin loop structure were ligated to prepare for hybridization. To select cDNA fragments of preferentially 150∼200 bp in length, the library fragments were purified with AMPure XP system (Beckman Coulter, Beverly, USA). Then 3 μl USER Enzyme (NEB, USA) was used with size-selected, adaptor ligated cDNA at 37°C for 15 minutes followed by 5 minutes at 95°C before PCR. Then PCR was performed with Phusion High-Fidelity DNA polymerase, Universal PCR primers and Index (X) Primer. Then, PCR products were purified (AMPure XP system) and library quality was assessed on the Agilent Bioanalyzer 2100 system. The clustering of the index-coded samples was performed on a cBot Cluster Generation System using PE Cluster Kit cBot-HS (Illumina). After cluster generation, the library preparations were sequenced on an Illumina platform and paired-end reads were generated. Raw data (raw reads) of FASTQ format were firstly processed by Novogene. In this step, clean data (clean reads) were obtained by removing reads containing adapter and poly-N sequences and reads with low quality from raw data. At the same time, Q20, Q30 and GC content of the clean data were calculated. All the downstream analyses were based on the clean data with high quality. Reference genome and gene model annotation files were downloaded from genome website browser (NCBI/UCSC/Ensembl) directly. Paired-end clean reads were mapped to the reference genome using HISAT2 software. Differential gene expression was determined using the R package edgeR^74^ utilizing trimmed mean of M-values^81^ normalization. The exact Test function was applied to obtain genes differentially expressed between any two conditions. A false discovery rate (FDR) value below 0.05 was considered as threshold for significant differential gene expression. FPKM normalized expression values were obtained for further analyses.

Gene set enrichment analysis (GSEA) was performed using the R-package clusterProfiler (v4.6.2)^75^ and MSigDB v2022.1.Mm gene sets,^82^ utilizing the fgsea algorithm. The exponent parameter was set to 0 for unweighted analyses of log2 fold change sorted gene lists from RNA-Seq data. Cell type deconvolution was carried out using the CIBERSORTx algorithm^76^ (https://cibersortx.stanford.edu/) For the assessment of fibroblast purity, a signature matrix was created using the pancreas single-cell RNA-seq dataset from the Tabula Muris project^71^ downloaded from the human cell atlas data portal (https://data.humancellatlas.org). Normalized sequencing counts of 32 cells per annotated cell type were extracted using the R-package Seurat v4.3.0^77^ and mouse gene symbols were added using the R-package biomaRt v2.52.0 using ENSEMBL v100 annotation.

For KPC mice derived fibroblast subtype determination, a fibroblast-enriched sc-RNAseq dataset from Elyada et al.^20^ was utilized. Data was kindly provided by the authors. as CellRanger output files along with cell type annotations. Cells observed to express less than 200 or more than 4000 genes as well as cells exhibiting more than 15% mitochondrial gene expression were filtered out. Normalization of expression values was carried out using the SCTransform (v2) function of the Seurat R-package. Normalized sequencing counts of 32 cells per annotated fibroblast type were extracted. Cell fractions were computed using using the created signature matrices and bulk RNAseq FPKM values generated as described above, utilizing B-mode batch correction, 32 samples per cell type and 1000 permutations for significance analysis.

#### Single-cell RNA-sequencing analyses

Publicly available single-cell RNA-seq data sets have been processed and analyzed utilizing the R-package Seurat v5.1.0.^77^ Single-cell RNA-seq data of human PDAC and healthy control samples, described in Peng et al.^30^ have been obtained from the Genome Sequence Archive https://ngdc.cncb.ac.cn/bioproject/browse/PRJCA001063. KPC (*Pdx1-Cre; lox-stop-lox-Kras*^*G12D/+*^*; lox-stop-lox-Trp53*^*R172H/+*^) mice pancreas sample data, described in Elyada et al.^20^ have been kindly provided by Dr. Lindsey Baker.

Data of pancreas samples from normal, KIC (*Ptf1a/p48-Cre; lox-stop-lox-Kras*^*G12D/+*^*; Ink4a*^*lox/lox*^), KPfC (*Pdx1-Cre; lox-stop-lox-Kras*^*G12D/+*^*; Trp53*^*lox/lox*^) and KPC (*Ptf1a/p48-Cre; lox-stop-lox-Kras*^*G12D/+*^*; lox-stop-lox-Trp53*^*R172H/+*^) mouse models as described in Hosein et al.,^29^ have been obtained from NCBI GEO (https://www.ncbi.nlm.nih.gov/geo/query/acc.cgi?acc=GSE125588).

First, single patients/samples have been processed independently. For improving data quality, cells expressing less than distinct 200 genes or whose overall mitochondrial gene expression exceeded 15% (Hosein, Elyada) or 20% (Peng), determined by genes listed in MitoCarta v3^83^ have been removed from further analyses. Genes expressed in less than 20 cells were discarded and multiplet cells have been detected and removed utilizing the R-package scDblFinder v1.18.0.^78^ Expression values have been normalized using Seurat's SCTransform v2 algorithm, employing the 2000 most variable genes and regressing for mitochondrial gene expression and cell cycle phase. Separate samples of the respective data sets have been integrated by applying the Seurat functions SelectIntegrationFeatures (nfeatures = 2000), PrepSCTIntegration, FindIntegrationAnchors, and IntegrateData. Cell clusters have been calculated using the Seurat functions FindNeighbors and FindClusters (Louvain algorithm). If provided with the data sets, cell type annotations have been used (Peng, Elyada), else cell clusters have been assigned cell identities by inspecting marker gene expression (Fibroblasts: pronounced expression of *Col1a1* and *Col1a2*).

#### Establishing ANGPTL4-overexpressing fibroblast cell line

To further investigate the functional role of the *ANGPTL4* gene in mediating crosstalk with preneoplastic cancer, we overexpressed *Angptl4* in an immortalized fibroblasts cell line (SV-80) using *ANGPTL4* CRISPR activation plasmid that was constructed by Santa Cruz Biotechnology.

#### Transfection and selection

An *ANGPTL4* CRISPR activation plasmid that was constructed by Santa Cruz Biotechnology (US) was used to create the overexpression model using SV-80 fibroblasts. The day before transfection of the activation and the control plasmid, 0.25×10^6^ cells per well were seeded into a 6-well plate. 2.5 μg of plasmid-DNA was carefully dissolved and mixed with 95 μl of Opti-MEM I Reduced Serum Medium (Thermo Fisher Scientific, Germany) and 5 μl of P3000 reagent. After dissolving Lipofectamin 3000 (Thermo Fisher Scientific, Germany) in Opti-MEM for 5 minutes at room temperature, the solution was mixed with the dissolved plasmid-DNA. Following 20 minutes of incubation 250 μl of the final solution were transferred into one well of a 6-well plate and incubated for 48 hours (at 37°C and 5% CO_2_). Next, 0.15 μg / ml Puromycin was used for selection. Serial and limited dilution approach was used to generate clones. Cell colonies of clones were selected under light microscopy. At least three clones from both the activated and control plasmid transfections were selected for further validation and analysis of the overexpression cell lines.

#### Western blot

To analyze the overexpression of ANGPTL4 expression on the protein level, a western blot was performed. Proteins were extracted from cultured cells using a lysis buffer containing 7 M urea, 2 M thiourea, 4% CHAPS, and 40 mM DTT. Protein levels were quantified using a BIORAD protein assay dye. The collected proteins were denatured through heating at 95°C. From each sample, 40 μg of protein was separated by 10% sodium dodecyl sulfate-polyacrylamide gel electrophoresis, transferred to polyvinylidene difluoride membranes (PVDF, GE Healthcare Life science, Great Britain), blocked for 1 hour in 5% milk+ TBS/T, and incubated with primary antibodies (ANGPTL4-1:1500) overnight at 4°C. The next day, the membranes were washed with Tris-buffered saline with 0.1% Tween-20 (TBS/T) and treated with suitable secondary antibodies (Goat anti-Rabbit (1:5000). Vinculin (1:1000) for ANGPTL4 was used as a loading control. Protein bands were visualized by an enhanced chemiluminescent substrate and quantified by ImageJ.

#### MTT assay

To quantify the proliferation of Angptl4-overexpressing SV-80 clones compared to control SV-80 clones and PANC-1 cell lines co-cultured with conditioned media from both types of clones, an MTT assay was performed. On day 1, cells were seeded in 96-well plates (2×10^3^ cells per well) with a growth medium specific to each cell type. After 24 and 72 hours of incubation at 37°C in 5% CO_2_, MTT solution was added and incubated for 4 hours. Following this, DMSO was added, and absorbance was measured at 570 nm after shaking. Additionally, PANC-1 cells exposed to 50 % conditioned media from both overexpressed and control clones were included to assess the role of fibroblast-mediated ANGPTL4 in affecting the proliferation of cancer cells.

#### Wound healing assay

To evaluate 2D cell migration, a wound healing assay was conducted comparing Angptl4-overexpressing SV-80 clones to control SV80 clones and PANC-1 cell lines co-cultured with conditioned media from both types of clones. Cells (3x10^6^) were seeded in a culture insert (ibidi 2-well insert, ibidi GmbH, Martinsried, Germany) with growth medium for 24 hours. After removal of the inserts, the cells were incubated in a serum-reduced medium. Wound closure was monitored by capturing images at 0, 2, 4, 6, and 8 hours, with wound width quantified using ImageJ and the Wound Healing Size Tool plugin^84^ Further, to investigate the role of fibroblast-mediated Angptl4 in cancer cell migration, PANC-1 cells were treated with 50% conditioned media from both overexpressed and control clones. Following a similar experimental setup, a wound-healing assay was also performed using KPC3595 cell lines exposed to varying doses (5 μg / ml, 10 μg / ml, and 20 μg / ml) of recombinant LAMA2 to quantify the migration of KPC3595 cells in the presence of different concentrations of LAMA2.

#### Migration assay

A transwell migration assay was performed using a 24-well Boyden chamber with 8 μm pore-size inserts to assess chemotactic movement. The 20,000 cells were seeded in serum-free DMEM in the upper chamber. The lower chamber contained the culture medium with 10% FBS. After incubation for 24 hours, cells on the top surface of the membrane were wiped off. The membrane was then stained with Hematoxylin at RT for 30 minutes. Cells on the bottom surface of the membrane were examined under a light microscope. Cells from 5 random fields were counted using ImageJ and averaged. Additionally, PANC-1 cells were seeded in the upper chamber, while control and ANGPTL4-overexpressing fibroblast clones were seeded in the lower chamber to assess the impact of fibroblast-mediated ANGPTL4 on cancer cell migration. Following a similar experimental setup, a transwell migration assay was also performed using KPC3595 cell lines exposed to varying doses (5 μg / ml, 10 μg / ml, and 20 μg / ml ) of recombinant LAMA2 to quantify the migration of KPC3595 cells in the presence of different concentrations of LAMA2.

#### Invasion assay

For the invasion assay, KPC3595 cells are seeded into the upper compartment of a transwell insert coated with a diluted matrigel. The Matrigel, prepared by diluting in ice-cold, serum-free DMEM to a final concentration of 1 mg / ml, is added (50 μl) to the insert and allowed to solidify at 37°C for 1 hour. After gel solidification, KPC cells (10,000) are seeded on top of the ECM layer in serum-free media, with recombinant LAMA2 added as a chemoattractant at concentrations of 5 μg / ml, 10 μg / ml, and 20 μg / ml in the well. A control group without Lama2 treatment was also included. The invasion was assessed by counting the number of cells that migrated through the ECM towards the LAMA2 in the lower chamber using ImageJ for quantification.

### Quantification and statistical analysis

#### Statistical analysis

Statistical analysis was performed using GraphPad Prism software, version 8.4 (GraphPad, San Diego, CA, USA). Data are shown as means ± standard deviation (s.d.), unless otherwise specified. For the primary isolated fibroblast RT-qPCR and co-culture experiments (Figures 7A, 9A, 12A, and 13A), statistical significance was determined using an unpaired two-tailed Student’s t-test with Welch’s correction. Quantification of ANGPTL4 overexpression by RT-qPCR (Figure 9B), MTT, and migration assays with ANGPTL4_OE colonies (Figures 9D, 9E, 10A, and 10B) was also analyzed using an unpaired two-tailed Student’s t-test. For MTT, invasion, and migration assays with LAMA2 recombinant protein (Figures 13B, 13C, and 13E), statistical significance was assessed using one-way ANOVA. For quantification of ANGPTL4 overexpression by Western blot (Figure 9C) and wound healing assays (Figures 9G, 10D, and 13F), statistical significance was assessed using two-way ANOVA. *P* < 0.05 was considered as significant.
