# Supplementary material for: Gene expression dynamics in fibroblasts during early-stage murine pancreatic carcinogenesis
Source: iScience. 2024 Dec 10;28(1):111572. doi: 10.1016/j.isci.2024.111572 (PMC11731286; doi:10.1016/j.isci.2024.111572)
Supplement: Document S1. Figures S1–S6 and Tables S1–S5 [file mmc1.pdf]

iScience, Volume *28*

Supplemental information

Gene expression dynamics in fibroblasts during early-stage murine pancreatic carcinogenesis

Nupur Ohri, Johanna Häußler, Nino Javakhishvili, David Vieweg, Anais Zourelidis, Bogusz Trojanowicz, Monika Haemmerle, Irene Esposito, Markus Glaß, Yoshiaki Sunami, and Jörg Kleeff

Supplemental legends

Supplementary Figure S1: Expression of 26 relevant DEGs in 19 different clusters.
UMAP plot based on scRNA-seq data from Hosein et al. showing distinct cell populations and the identification of 19 clusters, as previously presented in Figure 6B and 6C.

Supplementary Figure S2: Expression of 26 relevant DEGs in different stages and pancreatic cancer mouse models.
UMAP plot based on scRNA-seq data from Hosein et al. illustrating gene expression variations across different pancreatic cancer mouse models at distinct time points (early: 40 days, late: 60 days), as referred in Figure 6D.

Supplementary Figure S3: Expression of 26 relevant DEGs in different cell types.
Analysis of scRNA-seq data for KPC mice from Elyada et al. highlighting the expression of the 26 relevant DEGs across various cell types.

Supplementary Figure S4: Expression of 26 relevant DEGs in human tumor and non-tumor cells.
Evaluation of scRNA-seq data from Peng et al. focusing on the expression profiles of the 26 DEGs in human pancreatic tumor and non-tumor cells.

Supplementary Figure S5: Expression of 26 relevant DEGs in CAF subtypes.
Analysis of scRNA-seq data from Elyada et al. detaining the expression of the 26 DEGs in different CAF subtypes.

Supplementary Figure S6: Elevated FBLN2 and THBS1 expression in PDAC.
Immunohistochemistry results conducted on representative human pancreatic tissues (non-tumor and PDAC) demonstrating elevated expression levels of FBLN2 and THBS1.

Supplementary Table 1: FKPM values of key genes for RNA-seq data (4 weeks)
Supplementary Table 2: FKPM values of key genes for RNA-seq data (6 weeks)
Supplementary Table 3: FKPM values of key genes for RNA-seq data (8 weeks)
Supplementary Table 4: FKPM values of key genes for RNA-seq data (10 weeks)
Supplementary Table 5: FKPM values of key genes for RNA-seq data (12 weeks)

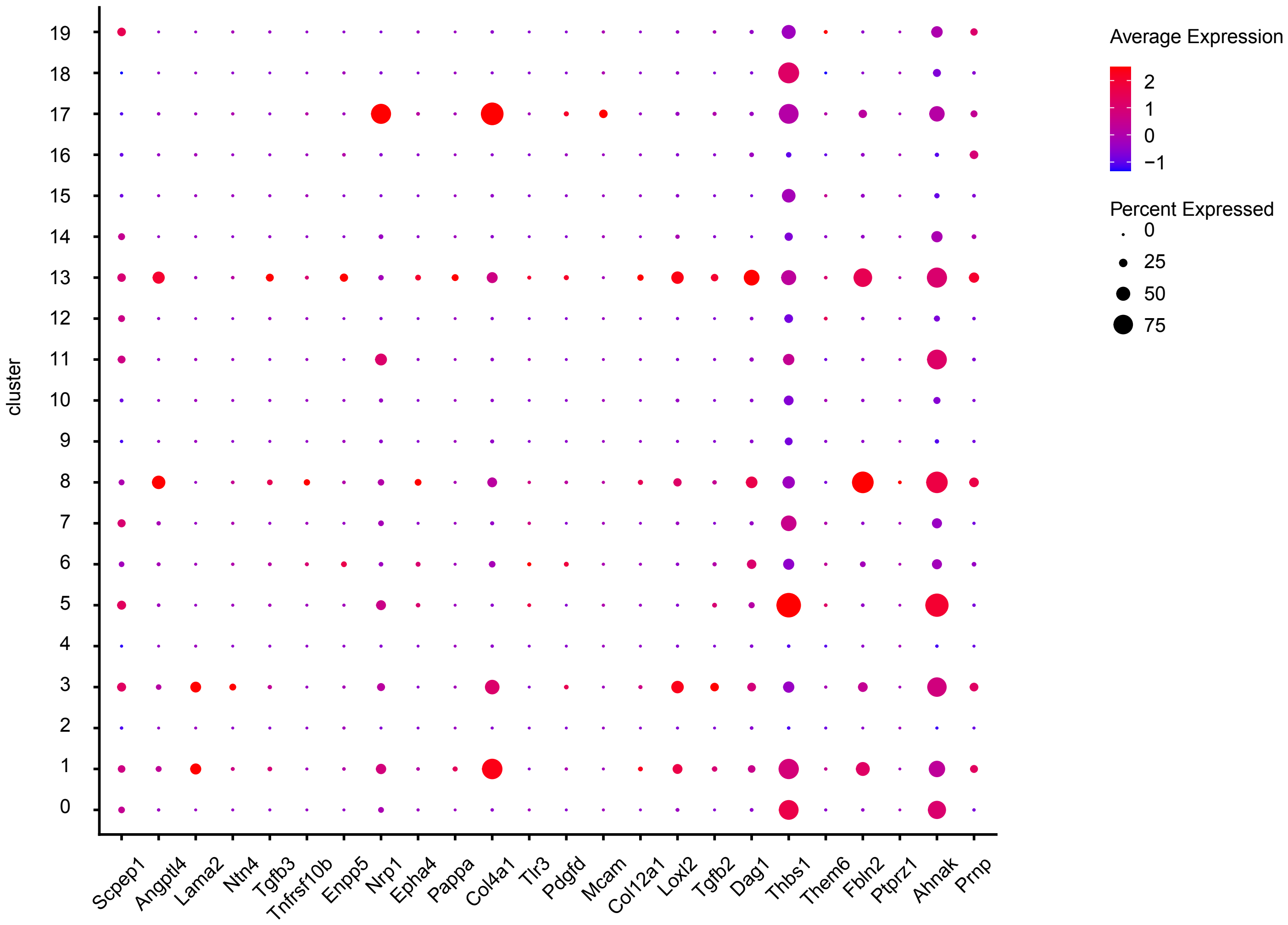
Average Expression
2
1
0
−1
Percent Expressed
0
25
50
75
cluster
19
18
17
16
15
14
13
12
11
10
9
8
7
6
5
4
3
2
1
0
Scpep1
Angptl4
Lama2
Ntn4
Tgfb3
Tnfrsf10b
Enpp5
Nrp1
Epha4
Pappa
Col4a1
Tlr3
Pdgfd
Mcam
Col12a1
Loxl2
Tgfb2
Dag1
Thbs1
Them6
Fbln2
Ptprz1
Ahnak
Prnp

Figure S1

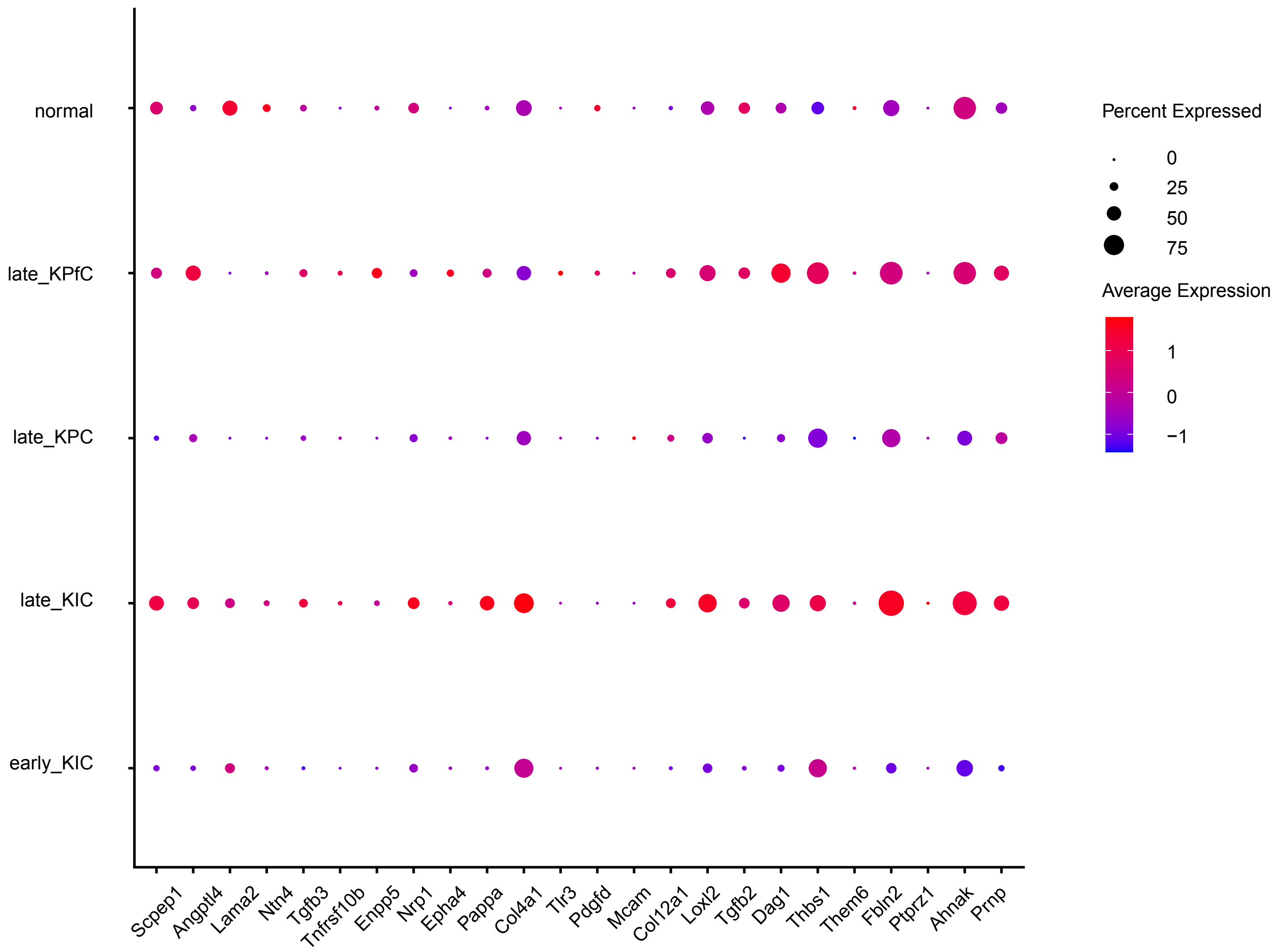
normal
late_KPfC
late_KPC
late_KIC
early_KIC
Scpep1
Angptl4
Lama2
Ntn4
Tgfb3
Tnfrsf10b
Enpp5
Nrp1
Epha4
Pappa
Col4a1
Tlr3
Pdgfd
Mcam
Col12a1
Loxl2
Tgfb2
Dag1
Thbs1
Them6
Fbln2
Ptprz1
Ahnak
Prnp
Percent Expressed
0
25
50
75
Average Expression
1
0
−1

Figure S2

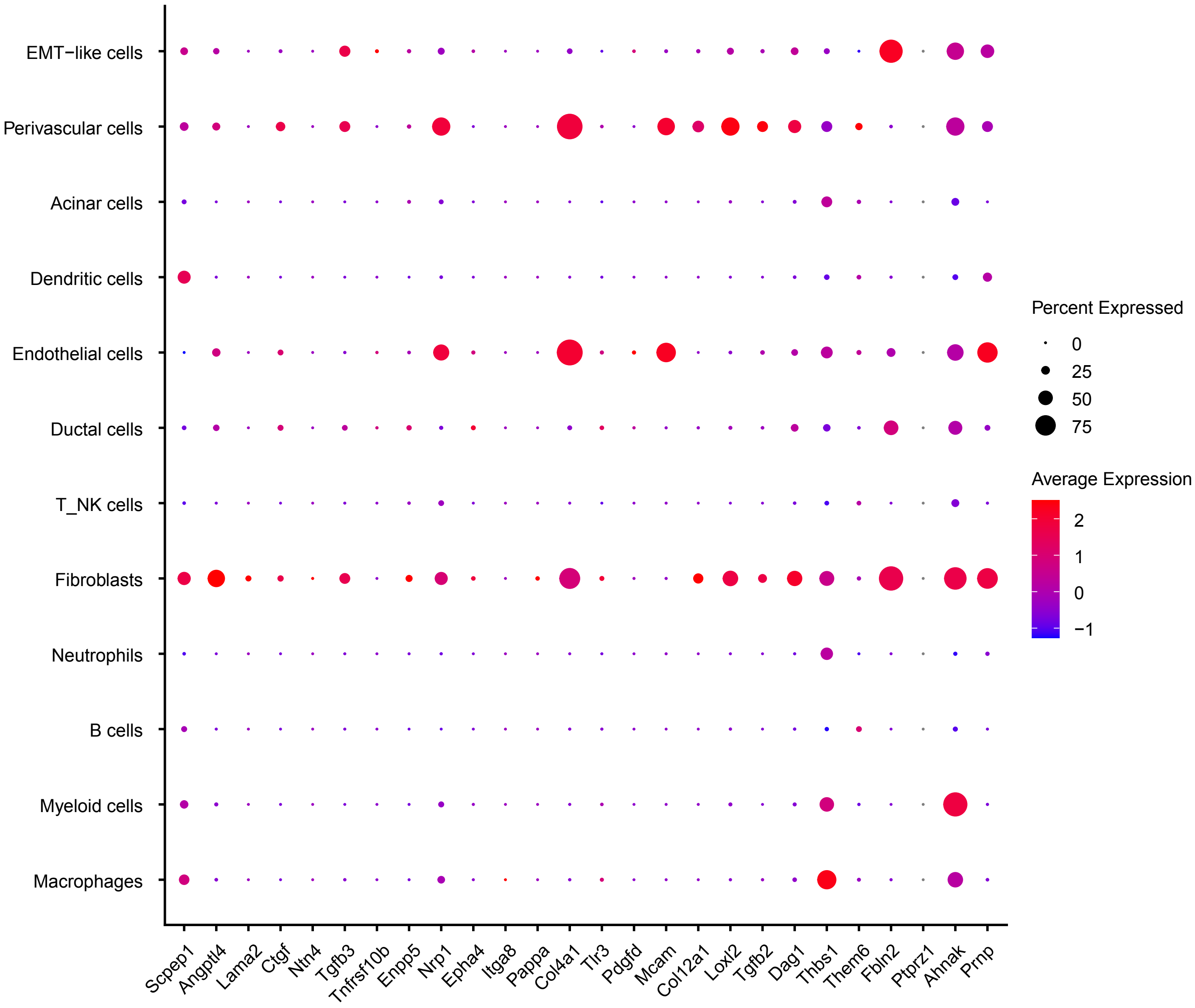
EMT−like cells
Perivascular cells
Acinar cells
Dendritic cells
Endothelial cells
Ductal cells
T_NK cells
Fibroblasts
Neutrophils
B cells
Myeloid cells
Macrophages
Scpep1
Angptl4
Lama2
Ctgf
Ntn4
Tgfb3
Tnfrsf10b
Enpp5
Nrp1
Epha4
Itga8
Pappa
Col4a1
Tlr3
Pdgfd
Mcam
Col12a1
Loxl2
Tgfb2
Dag1
Thbs1
Them6
Fbln2
Ptprz1
Ahnak
Prnp
Percent Expressed
0
25
50
75
Average Expression
2
1
0
−1

Figure S3

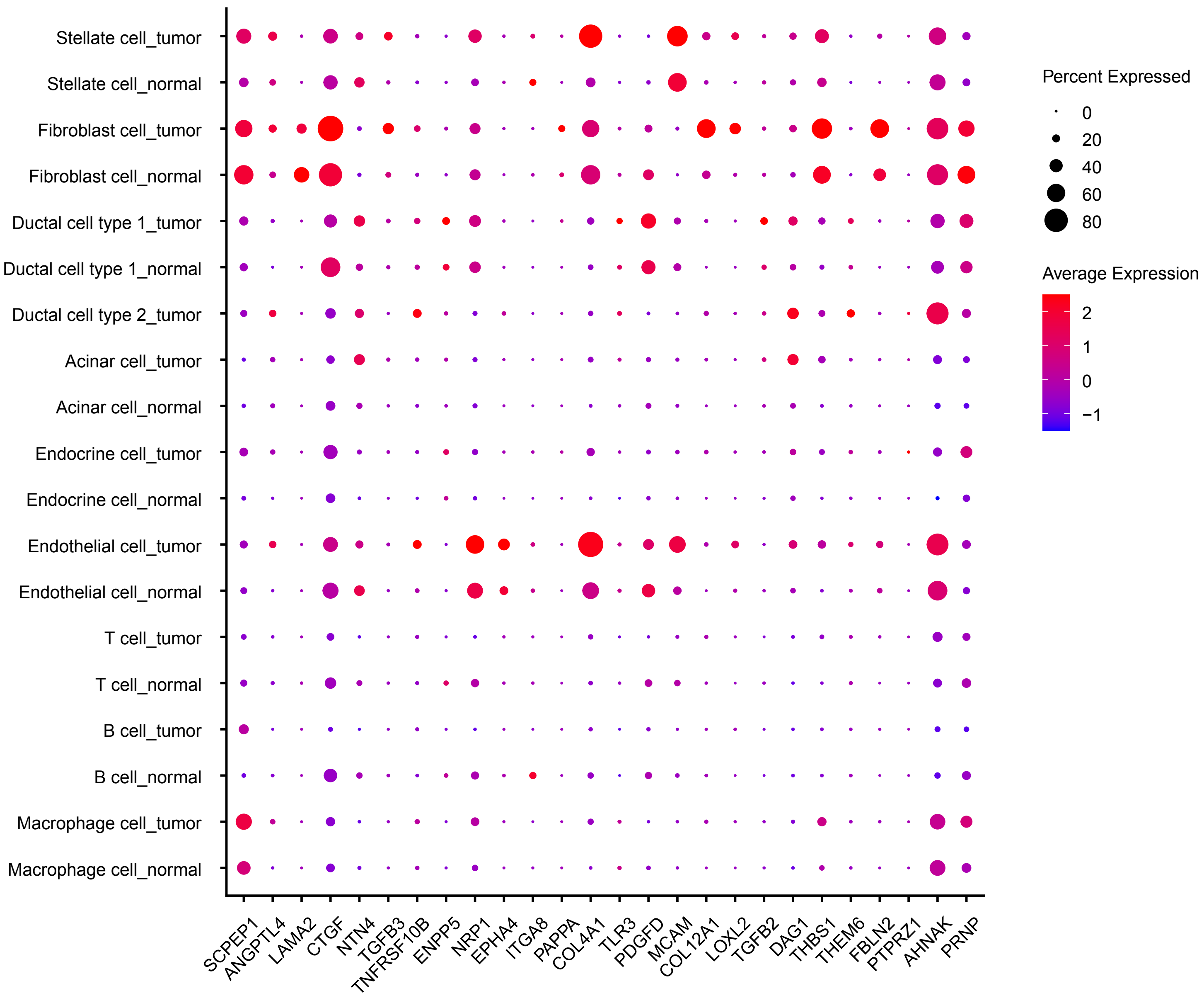
Stellate cell_tumor
Stellate cell_normal
Fibroblast cell_tumor
Fibroblast cell_normal
Ductal cell type 1_tumor
Ductal cell type 1_normal
Ductal cell type 2_tumor
Acinar cell_tumor
Acinar cell_normal
Endocrine cell_tumor
Endocrine cell_normal
Endothelial cell_tumor
Endothelial cell_normal
T cell_tumor
T cell_normal
B cell_tumor
B cell_normal
Macrophage cell_tumor
Macrophage cell_normal
SCPEP1
ANGPTL4
LAMA2
CTGF
NTN4
TGFB3
TNFRSF10B
ENPP5
NRP1
EPHA4
ITGA8
PAPPA
COL4A1
TLR3
PDGFD
MCAM
COL12A1
LOXL2
TGFB2
DAG1
THBS1
THEM6
FBLN2
PTPRZ1
AHNAK
PRNP
Percent Expressed
0
20
40
60
80
Average Expression
2
1
0
−1

Figure S4

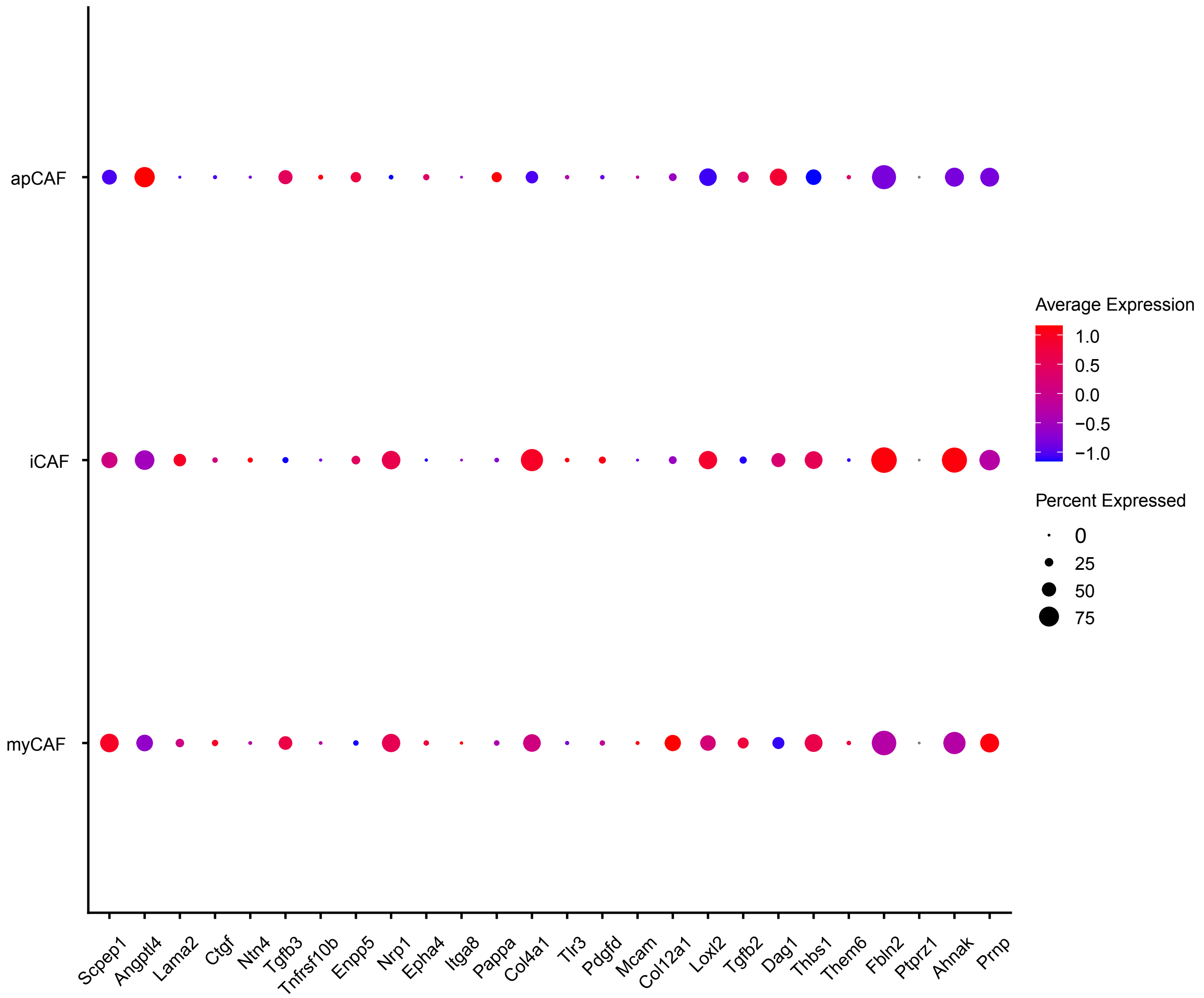
apCAF
iCAF
myCAF
Scpep1
Angptl4
Lama2
Ctgf
Ntn4
Tgfb3
Tnfrsf10b
Enpp5
Nrp1
Epha4
Itga8
Pappa
Col4a1
Tlr3
Pdgfd
Mcam
Col12a1
Loxl2
Tgfb2
Dag1
Thbs1
Them6
Fbln2
Ptprz1
Ahnak
Prnp
Average Expression
1.0
0.5
0.0
−0.5
−1.0
Percent Expressed
0
25
50
75

Figure S5

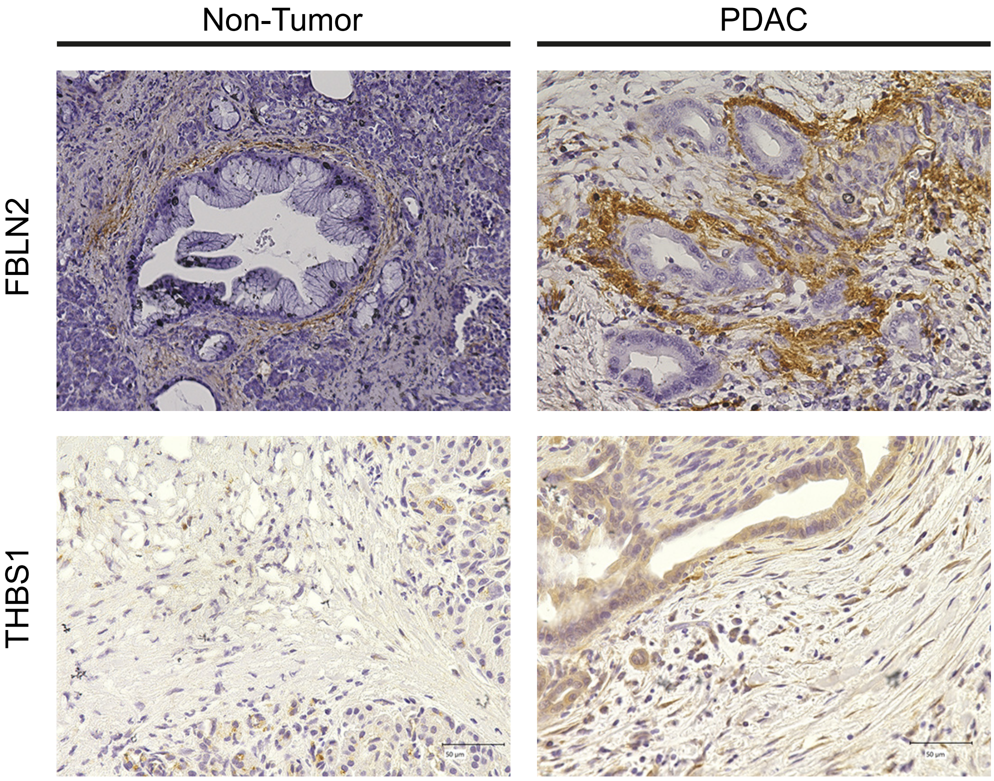

Figure S6

Supplementary Table 1: FPKM values of key genes for RNA-seq data (4 weeks)

	gene	chr	length	log2FC	pValue	FDR	Cre	Cre	Cre	Cre	KPC	KPC	KPC
ENSMUSG00000069833	Ahnak	19	19938	0.758779	0.042902	1	29.42096	31.98496	37.9943	33.3657	52.08419	62.18332	54.21867
ENSMUSG00000002289	Angptl4	17	3541	-0.62547	0.188822	1	8.87363	5.724456	5.696593	14.20456	5.196006	4.645571	6.933337
ENSMUSG00000032332	Col12a1	9	15228	0.304708	0.529789	1	104.5592	87.30434	115.2415	77.25909	107.1663	129.336	119.5635
ENSMUSG00000031502	Col4a1	8	7464	0.054891	0.865452	1	473.4706	481.4512	471.7553	286.79	354.6956	532.7198	447.5152
ENSMUSG00000019997	Ctgf	10	2861	0.367243	0.285175	1	682.3636	768.8875	825.4765	706.7591	870.6207	1165.237	850.4048
ENSMUSG00000039952	Dag1	9	6357	0.297374	0.282044	1	30.45618	35.53185	32.85004	25.8577	43.34179	35.60246	35.98427
ENSMUSG00000023960	Enpp5	17	4858	0.731496	0.179598	1	4.331777	12.12931	8.107447	11.04916	18.9801	11.93816	13.43641
ENSMUSG00000026235	Epha4	1	8054	-0.00027	0.983377	1	4.465084	2.060291	5.009098	1.309401	1.882856	4.362289	3.384867
ENSMUSG00000064080	Fbln2	6	6370	0.11563	0.707646	1	600.8999	630.1722	663.7914	742.375	486.1401	918.7011	738.1354
ENSMUSG00000026768	Itga8	2	7368	0.537866	0.415186	1	4.32328	9.02816	6.097262	2.288388	10.3136	6.977439	6.375734
ENSMUSG00000019899	Lama2	10	16605	0.306472	0.587974	1	3.326703	2.770689	4.124119	3.13179	2.380522	5.893312	4.110937
ENSMUSG00000034205	Loxl2	14	5405	0.579297	0.233743	1	155.2356	121.128	123.4797	70.17683	115.6999	226.3012	184.6941
ENSMUSG00000032135	Mcam	9	3350	0.55762	0.337358	1	60.07436	87.84543	80.44161	25.89118	105.7824	94.21632	80.66103
ENSMUSG00000025810	Nrp1	8	9785	-0.39892	0.3627	1	23.16699	15.35136	12.80567	16.20518	11.956	12.61004	13.84536
ENSMUSG00000020019	Ntn4	10	3741	0.140061	0.777235	1	36.7948	35.91156	34.18921	21.01598	36.19046	31.99029	37.53155
ENSMUSG00000028370	Pappa	4	11027	0.565163	0.454056	1	17.59048	8.476097	20.61523	2.571325	19.48542	22.169	13.00107
ENSMUSG00000032006	Pdgfd	9	3154	0.340029	0.611493	1	12.81864	9.617274	17.12713	8.599425	11.12106	19.80452	14.79656
ENSMUSG00000079037	Prnp	2	2343	0.48118	0.218341	1	74.60531	128.0783	98.56942	107.5262	175.1098	114.5385	138.3099
ENSMUSG00000068748	Ptprz1	6	14245	0.945045	0.082925	1	4.775846	9.237461	8.107488	2.941348	12.66255	13.54586	9.977577
ENSMUSG00000000278	Scpep1	11	2097	0.536328	0.231753	1	134.7189	231.7613	171.7124	188.0022	292.5665	214.2925	283.0255
ENSMUSG00000039239	Tgfb2	1	5791	0.179632	0.703718	1	16.09732	13.55848	17.9182	4.819885	15.13876	16.23205	13.13341
ENSMUSG00000021253	Tgfb3	12	3383	0.150461	0.774689	1	84.37014	87.94664	165.4685	40.38526	111.6544	129.196	73.95558
ENSMUSG00000040152	Thbs1	2	6073	0.063015	0.865735	1	1535.168	1074.556	1678.582	1362.98	914.7049	2144.048	1368.943
ENSMUSG00000056665	Them6	15	1784	-0.73159	0.155246	1	3.150943	4.284581	2.37638	2.8849	2.166279	1.561199	2.002964
ENSMUSG00000031639	Tlr3	8	5207	0.417218	0.447089	1	0.823512	1.31931	1.195014	1.024795	1.500537	1.459808	1.407989
ENSMUSG00000022074	Tnfrsf10b	14	5451	0.100662	0.744921	1	11.24447	10.48141	13.34076	14.16832	10.2956	18.27173	11.02532

Supplementary Table 2: FPKM values of key genes for RNA-seq data (6 weeks)

	gene	chr	length	log2FC	pValue	FDR	Cre	Cre	Cre	Cre	KPC	KPC	KPC	KPC
ENSMUSG00000069833	Ahnak	19	19938	-0.06185	0.860301	1	50.06325	47.11466	59.87579	42.43975	50.42027	37.83506	43.8122	59.05525
ENSMUSG00000002289	Angptl4	17	3541	-0.23422	0.586292	1	12.33415	5.682752	6.399675	7.877423	8.053323	6.052526	8.320546	5.034431
ENSMUSG00000032332	Col12a1	9	15228	0.176076	0.703508	1	233.4528	80.37121	165.6727	91.13383	127.263	181.5759	230.3385	105.5234
ENSMUSG00000031502	Col4a1	8	7464	-0.06756	0.833706	1	346.8458	365.6613	692.0342	411.4814	436.5624	348.1787	421.0991	527.0963
ENSMUSG00000019997	Ctgf	10	2861	0.31082	0.336314	1	537.1361	833.6481	1013.669	689.5432	694.3116	1019.202	1001.721	1097.764
ENSMUSG00000039952	Dag1	9	6357	0.256771	0.321909	1	45.49265	32.37428	47.68405	31.65288	48.47708	46.39563	41.86535	51.08802
ENSMUSG00000023960	Enpp5	17	4858	0.077666	0.88028	1	14.48449	11.35699	12.27464	11.0264	14.8526	13.56057	10.43868	13.0104
ENSMUSG00000026235	Epha4	1	8054	0.32053	0.609597	1	0.993239	2.769813	7.172084	2.736541	4.470213	0.606534	1.403023	10.59449
ENSMUSG00000064080	Fbln2	6	6370	-0.21278	0.469507	1	641.1502	736.9911	1024.105	653.7766	827.2609	507.4264	524.5942	777.6784
ENSMUSG00000026768	Itga8	2	7368	-0.26825	0.671218	1	3.061611	6.950832	10.8612	5.891719	7.526816	4.483912	3.057895	7.156214
ENSMUSG00000019899	Lama2	10	16605	0.654164	0.231691	1	0.559384	2.157972	5.159194	1.641626	3.953792	3.128958	4.078914	3.815435
ENSMUSG00000034205	Loxl2	14	5405	0.043145	0.925153	1	119.9733	177.6114	212.6165	122.1491	170.1079	156.1794	123.2712	201.9868
ENSMUSG00000032135	Mcam	9	3350	0.0052	0.992531	1	10.81922	66.96054	129.9988	55.23316	90.6675	13.64655	16.11026	143.5447
ENSMUSG00000025810	Nrp1	8	9785	-0.37784	0.34078	1	21.05434	15.9377	17.65092	19.88272	13.27308	11.17943	19.97916	12.92251
ENSMUSG00000020019	Ntn4	10	3741	0.192749	0.693357	1	34.93303	28.10718	54.49409	45.87829	18.78706	76.50674	65.04415	26.41558
ENSMUSG00000028370	Pappa	4	11027	-0.07273	0.920372	1	2.203857	12.66177	31.8979	6.610232	16.39644	6.778247	5.406671	22.16842
ENSMUSG00000032006	Pdgfd	9	3154	0.151697	0.816236	1	6.394884	11.75338	19.37112	7.189238	9.853492	17.20729	11.78127	10.81439
ENSMUSG00000079037	Prnp	2	2343	0.253416	0.490858	1	208.7535	168.9346	220.3864	142.3661	171.1097	245.8186	249.3238	216.3541
ENSMUSG00000068748	Ptprz1	6	14245	0.01134	0.982589	1	4.516501	6.34227	13.18198	7.087491	6.818156	7.310537	5.618499	11.62553
ENSMUSG00000000278	Scpep1	11	2097	0.037394	0.929602	1	209.3596	231.7766	215.0283	205.2115	157.6905	353.779	232.2564	140.2421
ENSMUSG00000039239	Tgfb2	1	5791	-0.42771	0.350464	1	12.26871	12.51786	20.2795	17.7082	9.653055	10.01539	7.936862	19.06255
ENSMUSG00000021253	Tgfb3	12	3383	0.727779	0.164392	1	17.77395	82.27476	226.4509	73.01965	57.4338	298.5392	186.1402	119.4953
ENSMUSG00000040152	Thbs1	2	6073	0.337059	0.368949	1	1762.499	1294.693	2007.153	1345.723	2042.966	1909.334	2253.691	1891.068
ENSMUSG00000056665	Them6	15	1784	-0.24726	0.597148	1	3.315221	3.282149	2.739754	3.617344	2.539893	2.944007	3.186455	2.239372
ENSMUSG00000031639	Tlr3	8	5207	-0.12633	0.811738	1	1.951327	1.251222	1.228821	1.083593	1.891755	0.824284	0.672346	1.672013
ENSMUSG00000022074	Tnfrsf10b	14	5451	-0.42678	0.149921	1	12.4219	13.95677	15.06397	11.6318	8.746246	10.5054	10.1361	10.08775

Supplementary Table 3: FPKM values of key genes for RNA-seq data (8 weeks)

	gene	chr	length	log2FC	pValue	FDR	Cre	Cre	Cre	Cre	KPC	KPC	KPC	KPC
ENSMUSG00000069833	Ahnak	19	19938	-0.24603	0.484015	1	52.73377	40.024	66.25053	44.34237	45.19539	43.06265	39.5872	43.62214
ENSMUSG00000002289	Angptl4	17	3541	-0.01569	0.97191	1	5.377253	16.227	4.420276	4.974722	8.833765	4.77732	4.21165	12.84511
ENSMUSG00000032332	Col12a1	9	15228	-0.08327	0.857129	1	135.8353	93.83424	145.1302	110.5086	135.8581	145.1506	93.77792	83.30228
ENSMUSG00000031502	Col4a1	8	7464	-0.30382	0.345513	1	635.0181	437.1314	603.0092	401.7192	363.7998	583.4997	435.5336	299.6654
ENSMUSG00000019997	Ctgf	10	2861	-0.1773	0.583129	1	932.1187	794.0345	931.6749	934.9845	666.5191	785.1899	789.4963	936.1097
ENSMUSG00000039952	Dag1	9	6357	0.051087	0.843856	1	39.24503	31.83023	44.84932	34.53715	37.38677	45.50768	34.24535	38.74354
ENSMUSG00000023960	Enpp5	17	4858	-0.59744	0.247314	1	11.20999	7.966439	10.75934	17.37781	9.236595	8.231399	5.820802	7.97737
ENSMUSG00000026235	Epha4	1	8054	-0.77183	0.220826	1	5.474658	3.024847	7.273052	2.664696	2.9618	3.026577	3.203171	1.60647
ENSMUSG00000064080	Fbln2	6	6370	-0.3948	0.180073	1	1141.154	571.4525	1127.694	836.5024	742.2062	1087.261	546.9593	420.1372
ENSMUSG00000026768	Itga8	2	7368	-1.4782	0.021714	1	7.59403	4.227566	9.282758	9.33125	3.071165	1.134139	3.12813	3.587162
ENSMUSG00000019899	Lama2	10	16605	-0.71072	0.19397	1	4.512417	3.713999	5.421491	2.745069	1.968172	3.684715	1.805592	2.557828
ENSMUSG00000034205	Loxl2	14	5405	-0.32556	0.478698	1	253.6732	135.0589	210.4686	195.1337	171.1823	268.2641	127.1523	67.27669
ENSMUSG00000032135	Mcam	9	3350	-0.98116	0.078232	1	106.732	60.44298	102.7905	68.34445	47.60726	51.08104	55.00546	17.68742
ENSMUSG00000025810	Nrp1	8	9785	0.435443	0.272414	1	17.50334	13.40903	18.73484	18.33736	22.59573	21.0416	25.94556	22.35313
ENSMUSG00000020019	Ntn4	10	3741	-0.43041	0.379083	1	33.57862	30.20728	35.88063	49.15647	26.93844	17.56465	34.78081	31.14356
ENSMUSG00000028370	Pappa	4	11027	-0.89282	0.222705	1	25.10857	4.061984	25.41233	14.9027	11.67402	17.09687	6.03157	2.619827
ENSMUSG00000032006	Pdgfd	9	3154	-1.00896	0.124653	1	13.18673	10.70353	16.7527	20.97057	8.140716	10.19712	5.453041	6.82085
ENSMUSG00000079037	Prnp	2	2343	-0.19413	0.597557	1	171.049	88.1736	162.8198	265.0674	171.6735	167.0867	127.1534	134.6708
ENSMUSG00000068748	Ptprz1	6	14245	-1.13746	0.028478	1	8.496745	5.717029	14.15102	12.22609	3.705707	4.134924	3.442617	7.164874
ENSMUSG00000000278	Scpep1	11	2097	-0.44144	0.297247	1	273.3	170.6958	232.088	367.305	187.2858	171.6229	189.6358	219.7901
ENSMUSG00000039239	Tgfb2	1	5791	-0.55249	0.228279	1	22.81406	10.15063	21.77022	16.41464	14.4626	13.23325	14.45502	6.363086
ENSMUSG00000021253	Tgfb3	12	3383	-0.32177	0.537032	1	168.1794	86.75551	137.708	92.085	72.93528	78.51293	99.69744	136.676
ENSMUSG00000040152	Thbs1	2	6073	-0.1192	0.750448	1	1855.376	1702.265	2114.162	1290.543	1422.702	1968.815	1465.088	1553.608
ENSMUSG00000056665	Them6	15	1784	0.058476	0.900315	1	2.313117	3.394465	1.834491	2.874417	2.100919	2.2849	3.886652	2.584036
ENSMUSG00000031639	Tlr3	8	5207	-0.12423	0.815623	1	1.441495	1.183049	1.211506	1.322213	1.412707	1.612656	1.10845	0.602025
ENSMUSG00000022074	Tnfrsf10b	14	5451	-0.17359	0.55761	1	15.76661	13.1078	15.30562	12.93514	10.90501	12.82478	11.94563	14.96328

Supplementary Table 4: FPKM values of key genes for RNA-seq data (10 weeks)

	gene	chr	length	log2FC	pValue	FDR	Cre	Cre	KPC	KPC	KPC
ENSMUSG00000069833	Ahnak	19	19938	-0.56401	0.202657	1	84.22953	39.7385	24.37047	38.76455	62.64668
ENSMUSG00000002289	Angptl4	17	3541	0.646561	0.263992	1	5.928618	9.936321	7.683934	18.3982	11.17854
ENSMUSG00000032332	Col12a1	9	15228	-0.66558	0.250043	1	174.4754	127.7024	57.92241	119.3177	108.5144
ENSMUSG00000031502	Col4a1	8	7464	-0.47531	0.241492	1	506.6981	400.6302	228.004	407.6092	343.3643
ENSMUSG00000019997	Ctgf	10	2861	-1.22283	0.002822	1	1122.198	1100.455	298.3826	449.4822	680.5585
ENSMUSG00000039952	Dag1	9	6357	-0.29953	0.360077	1	45.77072	45.4409	31.83195	41.09963	38.23619
ENSMUSG00000023960	Enpp5	17	4858	-0.42665	0.499782	1	13.62537	17.31093	10.30371	12.08044	12.1402
ENSMUSG00000026235	Epha4	1	8054	-2.21385	0.005703	1	8.842081	3.555696	2.084647	0.802242	1.119335
ENSMUSG00000064080	Fbln2	6	6370	-0.96727	0.009453	1	1123.615	855.7469	425.6619	371.3498	721.574
ENSMUSG00000026768	Itga8	2	7368	-2.62952	0.001268	1	8.081048	7.033986	0.592046	1.991272	1.079604
ENSMUSG00000019899	Lama2	10	16605	-1.30456	0.057263	1	3.59936	2.731945	0.4328	0.67289	2.739674
ENSMUSG00000034205	Loxl2	14	5405	-1.27056	0.028383	1	255.6686	222.5228	72.04379	84.70325	140.5681
ENSMUSG00000032135	Mcam	9	3350	-1.99123	0.004621	1	77.76573	72.01947	22.70797	20.87507	12.92398
ENSMUSG00000025810	Nrp1	8	9785	0.595449	0.263442	1	19.2397	13.50245	17.06245	34.71254	22.433
ENSMUSG00000020019	Ntn4	10	3741	-1.51038	0.014576	1	40.47897	24.9862	8.153968	5.629926	20.68595
ENSMUSG00000028370	Pappa	4	11027	-1.57803	0.083611	1	28.84426	19.37822	8.153756	11.75458	4.317901
ENSMUSG00000032006	Pdgfd	9	3154	-1.00796	0.213287	1	13.29779	11.27605	9.12427	4.312228	4.887959
ENSMUSG00000079037	Prnp	2	2343	-0.70129	0.130271	1	339.9772	213.7695	98.51466	152.8001	259.5409
ENSMUSG00000068748	Ptprz1	6	14245	-2.39735	0.000279	0.647431	10.3669	9.894647	1.037645	2.734014	1.996974
ENSMUSG00000000278	Scpep1	11	2097	-0.65544	0.216675	1	254.7	231.0921	144.148	142.0582	176.424
ENSMUSG00000039239	Tgfb2	1	5791	-0.86493	0.132296	1	15.92253	14.74074	9.944271	10.46084	4.846849
ENSMUSG00000021253	Tgfb3	12	3383	-2.109	0.001524	1	106.1757	126.6775	19.45301	40.20327	21.30746
ENSMUSG00000040152	Thbs1	2	6073	-1.91185	6.40E-05	0.340327	3178.027	1877.737	322.6688	687.8229	1004.88
ENSMUSG00000056665	Them6	15	1784	1.611442	0.011591	1	2.00352	2.036152	11.2056	3.6618	3.65198
ENSMUSG00000031639	Tlr3	8	5207	-0.57925	0.372308	1	2.111447	1.184328	1.398269	0.918662	0.989341
ENSMUSG00000022074	Tnfrsf10b	14	5451	-0.83492	0.026019	1	16.56707	13.35106	6.954745	7.177493	11.02797

Supplementary Table 5: FPKM values of key genes for RNA-seq data (12 weeks)

	gene	chr	length	log2FC	pValue	FDR	Cre	Cre	Cre	KPC	KPC	KPC
ENSMUSG00000069833	Ahnak	19	19938	-1.20429	0.003458	0.036191	87.92739	39.85675	72.23797	33.77802	45.62318	7.392824
ENSMUSG00000002289	Angptl4	17	3541	1.63166	0.001375	0.017399	3.734742	6.651665	3.631643	10.09353	11.20693	22.17078
ENSMUSG00000032332	Col12a1	9	15228	-2.29372	4.33E-05	0.001077	213.8505	199.4646	130.4808	52.61909	13.62238	44.66051
ENSMUSG00000031502	Col4a1	8	7464	-1.38097	0.000267	0.004693	608.6395	730.0757	557.178	143.9735	143.2819	440.7428
ENSMUSG00000019997	Ctgf	10	2861	-1.27546	0.00077	0.011031	1093.842	2111.56	893.5299	540.1393	308.511	844.6399
ENSMUSG00000039952	Dag1	9	6357	-1.32313	1.42E-05	0.000434	57.21695	49.73056	40.80746	23.35947	10.67144	25.01962
ENSMUSG00000023960	Enpp5	17	4858	-2.54588	5.31E-05	0.001253	18.8869	15.8823	15.43428	5.325199	3.215904	0
ENSMUSG00000026235	Epha4	1	8054	-4.21463	3.88E-07	2.17E-05	9.899983	9.306354	4.805638	0.438486	0.705369	0.140455
ENSMUSG00000064080	Fbln2	6	6370	-1.31559	0.00014	0.002794	1225.767	1378.406	1002.23	582.7882	420.5697	445.5271
ENSMUSG00000026768	Itga8	2	7368	-5.74223	1.59E-10	3.16E-08	9.570544	12.41131	9.465757	0.386914	0.10205	0.076766
ENSMUSG00000019899	Lama2	10	16605	-3.74253	1.06E-07	7.32E-06	4.371646	8.276057	5.731393	0.471486	0.799977	0.095375
ENSMUSG00000034205	Loxl2	14	5405	-1.58574	0.003552	0.036906	200.902	314.8645	193.6618	104.9753	113.1911	18.14558
ENSMUSG00000032135	Mcam	9	3350	-2.10506	0.001571	0.019342	58.19224	127.6186	85.15164	10.30068	16.28499	36.43545
ENSMUSG00000025810	Nrp1	8	9785	1.42946	0.002202	0.02542	20.68729	13.05149	16.08743	24.66409	92.12546	17.42208
ENSMUSG00000020019	Ntn4	10	3741	-1.64462	0.004641	0.045379	43.60788	54.83759	35.09151	5.357016	4.734424	32.68779
ENSMUSG00000028370	Pappa	4	11027	-3.00278	0.000952	0.013008	31.21691	37.43358	18.65534	3.152495	1.659227	6.083379
ENSMUSG00000032006	Pdgfd	9	3154	-3.29121	6.15E-05	0.001404	13.51248	27.04179	13.00205	5.274786	0.105954	0
ENSMUSG00000079037	Prnp	2	2343	-1.23726	0.004132	0.041442	286.6055	285.5988	225.7095	144.4997	49.24254	144.6978
ENSMUSG00000068748	Ptprz1	6	14245	-2.82976	8.90E-06	0.000293	4.333553	17.9224	11.97979	0.480898	1.196428	3.152649
ENSMUSG00000000278	Scpep1	11	2097	-1.86435	0.000222	0.004051	292.1013	208.0752	227.0587	123.2238	71.47317	4.855027
ENSMUSG00000039239	Tgfb2	1	5791	-1.69562	0.001845	0.022064	14.41045	16.99084	16.71618	5.510582	2.957463	6.387659
ENSMUSG00000021253	Tgfb3	12	3383	-2.40016	0.000154	0.003019	89.95372	102.1475	112.9357	30.49998	23.68294	3.544471
ENSMUSG00000040152	Thbs1	2	6073	-3.59752	6.53E-14	3.78E-11	3544.42	4329.489	1967.624	452.4785	191.205	169.2827
ENSMUSG00000056665	Them6	15	1784	2.12107	0.000132	0.002645	2.137314	2.279596	2.089709	5.34248	6.36888	16.74004
ENSMUSG00000031639	Tlr3	8	5207	-1.74427	0.00477	0.046414	1.992906	1.063062	1.649138	0.996924	0.352983	0
ENSMUSG00000022074	Tnfrsf10b	14	5451	-1.01337	0.003365	0.035402	13.88372	14.7555	15.42275	8.836087	10.19212	2.739336
